# Conservation and Occurrence of Trans-Encoded sRNAs in the Rhizobiales

**DOI:** 10.3390/genes2040925

**Published:** 2011-11-18

**Authors:** Jan Reinkensmeier, Jan-Philip Schlüter, Robert Giegerich, Anke Becker

**Affiliations:** 1 Center for Biotechnology (CeBiTec), Bielefeld University, Universitätsstraße 27, 33615 Bielefeld, Germany; E-Mails: jreinken@cebitec.uni-bielefeld.de (J.R.); robert@techfak.uni-bielefeld.de (R.G.); 2 Institute of Biology III, Faculty of Biology, Albert-Ludwigs-University Freiburg, Schanzlestraße 1, 79102 Freiburg, Germany; E-Mail: jan-philip.schlueter@biologie.uni-freiburg.de

**Keywords:** trans-encoded sRNAs, comparative analyses, Rhizobiales

## Abstract

Post-transcriptional regulation by trans-encoded sRNAs, for example via base-pairing with target mRNAs, is a common feature in bacteria and influences various cell processes, e.g., response to stress factors. Several studies based on computational and RNA-seq approaches identified approximately 180 trans-encoded sRNAs in *Sinorhizobium meliloti*. The initial point of this report is a set of 52 trans-encoded sRNAs derived from the former studies. Sequence homology combined with structural conservation analyses were applied to elucidate the occurrence and distribution of conserved trans-encoded sRNAs in the order of Rhizobiales. This approach resulted in 39 RNA family models (RFMs) which showed various taxonomic distribution patterns. Whereas the majority of RFMs was restricted to *Sinorhizobium* species or the *Rhizobiaceae*, members of a few RFMs were more widely distributed in the Rhizobiales. Access to this data is provided via the RhizoGATE portal [1,2].

## 1. Introduction

In the past two decades the appreciation of small noncoding RNAs (sRNAs) and their importance rose from the status of an exceptional occurrence to that of a general and ubiquitous feature of gene regulation in prokaryotic and eukaryotic life. sRNAs were characterized to be involved in several cellular processes, e.g., response to a variety of cell stresses, regulation of quorum sensing, and toxin antitoxin systems [3–5]. Depending on their location and perfect or imperfect sequence complementarity to specific mRNA targets two major classes were determined: (i) cis-encoded sRNAs, located in antisense to their target mRNA and thus possessing perfect sequence complementarity, and (ii) trans-encoded sRNAs located independently from potential targets, commonly in intergenic regions (IGRs), where sequence complementarity to a possible target can be imperfect or disrupted [6]. Noncoding transcripts, generally 50–250 nt in length, act as (i) activator or repressor of translation (OxyS/fhlA; DsrA/rpoS), are involved in (ii) mRNA stabilization or degradation (GadY/GadX; RyhB/sodB), or act as (iii) target mimicry (6S RNA/CsrB and CsrC) [6–14].

### 1.1. *In Silico* Prediction of sRNAs

RNA functional analyses revealed the relevance of RNA secondary structure for their function. Including RNA secondary structure information, various bioinformatics approaches were developed to identify and analyze sRNAs. *In silico* screens were performed in several bacteria. In *Escherichia coli*, initially four comprehensive analyses of IGRs were conducted, based on comparative sequence- and secondary structure-analyses as well as promoter and terminator predictions. Several hundred sRNA candidates were identified and 36 validated experimentally [15–18]. Following these studies, dozens of sRNA candidates were predicted in other bacteria using similar approaches, e.g., in *Helicobacter pylori* [19], *Pseudomonas aeruginosa* [20], *Nitrosomonas europaea* [21]. Tools for de novo sRNA gene finding, such as RNAz [22] and EvoFold [23] use multiple genome alignments and focus on sequence and structure conservation. In contrast, the application of cmsearch [24] requires prior knowledge about a family of related sRNAs in different species. Scans with cmsearch base on a combination of HMMs and covariance models. Agreement between approaches is low, and a potentially large number of false positives is predicted [25]. As validation of candidates by experimental methods is usually required anyway, researchers have increasingly turned towards experimental screens.

### 1.2. Experimental Screens

High throughput studies based on the deep sequencing and tiling array technologies elevated the potential of sRNA identification enormously. Transcriptome studies of e.g., *H. pylori*, *Caulobacter crescentus*, and *Synechocystis sp*. PCC6803 revealed hundreds of new sRNAs [26–28]. In the order of Rhizobiales, transcriptome analyses, focused on sRNA identification, were reported for *Rhizobium etli*, *Agrobacterium tumefaciens*, and *S. meliloti* 1021. A tiling array study of the *R. etli* transcriptome resulted in identification of 17 putative trans-encoded sRNAs and 49 cis-encoded antisense sRNAs [29,30]. A deep sequencing approach in *A. tumefaciens* C58 identified 228 sRNA transcripts, 22 of which were experimentally confirmed via Northern blot experiments [31]. Beside individually detected and characterized sRNAs in *S. meliloti*, e.g., IncA, tmRNA, 4.5S RNA, and RNase P, bioinformatics based studies identified a set of sRNAs which were further validated by Northern blot experiments [32–37]. Recently, a comprehensive deep sequencing approach combined with microarray analyses extended the number of trans-encoded sRNAs to approximately 180 [38].

An experimental screen delivers *bona fide* sRNA transcripts, with no obvious hints towards a potential functional role. By necessity, it starts from a single species and does not by itself incorporate phylogenetic information. Hence, it calls for a subsequent *in silico* study where the transcripts obtained for one species are taken as pivot elements to study their conservation and distribution in larger phylogenetic units. One intrinsic limitation of this approach is clear: an sRNA widely distributed, e.g., in the Rhizobiales, but lacking in *S. meliloti*, cannot be found. Hence, a complete survey of the phylogenetic order or even class from a single pivot organism is not possible.

### 1.3. Overview of the Present Study

The present study starts from *S. meliloti* 1021 as the pivot organism and from 52 trans-encoded sRNA transcripts obtained in our aforementioned study [38]. For each transcript, we performed homology searches and constructed RNA family models (RFMs). Our goals are twofold:

- We want to increase our knowledge about the distribution pattern of potential sRNAs conserved in the Rhizobiales;
- We want to automate the bioinformatics steps that are necessary for RFM construction, as far as it is possible utilizing present-day bioinformatics tools.

The present article describes the RFM construction process, and discusses our observations made when applying these models to the Rhizobiales.

### 1.4. Our Pivot Organism and Its Kind Relation

The endosymbiont *S. meliloti* exists in two different life forms, either in a free-living state as a soil bacterium or in a symbiotic relationship with its leguminous host plants, e.g., *Medicago sativa*. In response to flavonoids secreted by the host plant *S. meliloti* induces the formation of root nodules. These are colonized by the bacteria which inside the nodules differentiate to endosymbiotic bacteroids that are capable of nitrogen fixation. Bacteroids support the plant with ammonia and in turn receive C4-metabolites, e.g., succinate, from the host [39].

The genome of *S. meliloti* consists of three replicons, a single chromosome (3.65 Mbp) and two megaplasmids pSymA (1.35 Mbp) and pSymB (1.68 Mbp). The chromosome encodes 3,351 genes predominantly involved in housekeeping functions. The 1,293 genes on megaplasmid pSymA encode, among other functions, the symbiotic apparatus. pSymB carries 1,583 genes mainly involved in exopolysaccharide synthesis and transporter functions [40–42].

Within the order of Rhizobiales, sequenced plant symbionts include *Mesorhizobium loti*, *Sinorhizobium fredii*, *R. etli*, and *Sinorhizobium medicae*. The order of Rhizobiales also comprises completely sequenced human-, animal- as well as plant-pathogens. The animal pathogen *B. melitensis*, for example, generally infects sheep and goats, but can act as a human pathogen as well [43]. *Bartonella henselae* is responsible for the cat-scratch disease of humans [44]. A well studied plant-pathogen is *A. tumefaciens*, which infects several dicotyledons and acts as powerful tool in plant genetics [45].

## 2. Results and Discussion

### 2.1. From sRNA Transcripts to Family Models

We define our notion of RNA family models and give an informal overview of how they are constructed, before we proceed to report on the findings obtained with these models. Details of family model construction are presented in the Methods section.

#### 2.1.1. RNA Family Models: Terminology

The deep sequencing approach by Schlüter *et al.* [38] elucidated the existence of approximately 1,100 noncoding transcripts encoded on the *S. meliloti* genome, about 180 of which were trans-encoded. Due to the presumed function as regulatory sRNAs, a subset of 52 trans-encoded transcripts was chosen for a first comparative study (see Section 3.1). Our pivotal transcripts are named *SmelXnnn*, consistent with Schlüter *et al.* [38], where *X* ∈ {*A*, *B*, *C*} denotes the location on pSymA, pSymB, or chromosome, respectively. Potentially related sRNAs found by candidate search are simply named RNA1, RNA2, *etc*.

For all transcripts, we constructed RNA family models. Informally speaking, an RFM is a set of related sRNAs combined with a method to scan genomes to search for additional family members. Creating RNA family models is not a fully automated process, but requires both high computational effort and human curation.

We constructed two types of RFMs:

- *Covariance models* (CMs) are stochastic models, capturing sequence and structure conservation in an alignment of family members. CMs can be automatically constructed by infernal [24], given such an alignment;
- *Thermodynamic matchers* (TDMs) are RNA folding programs, based on the established thermodynamic model, but tailored to a specific structural motif [46]. Production of such matchers is supported by the graphical editor Locomotif [47].

Both approaches to RFM construction are complementary. When sequence conservation is high enough such that a trustworthy multiple sequence alignment and consensus structure can be established, CMs can be constructed automatically. TDMs are appropriate if sequence conservation is much weaker than structure conservation, such that no candidates are found by sequence similarity search, or they cannot be aligned well. TDMs focus on structure and folding energy; they can ignore sequence conservation in some parts, e.g., in helices, and yet insist on conserved sequence motifs elsewhere, e.g., in loops. Building such a matcher requires human design decisions and some experimentation, and hence, it is more laborious. In this study, we constructed CMs as a rule and TDMs for selected families of special interest to promote identification of further family members.

#### 2.1.2. Overview of the Model Construction Process

**Figure 1:**
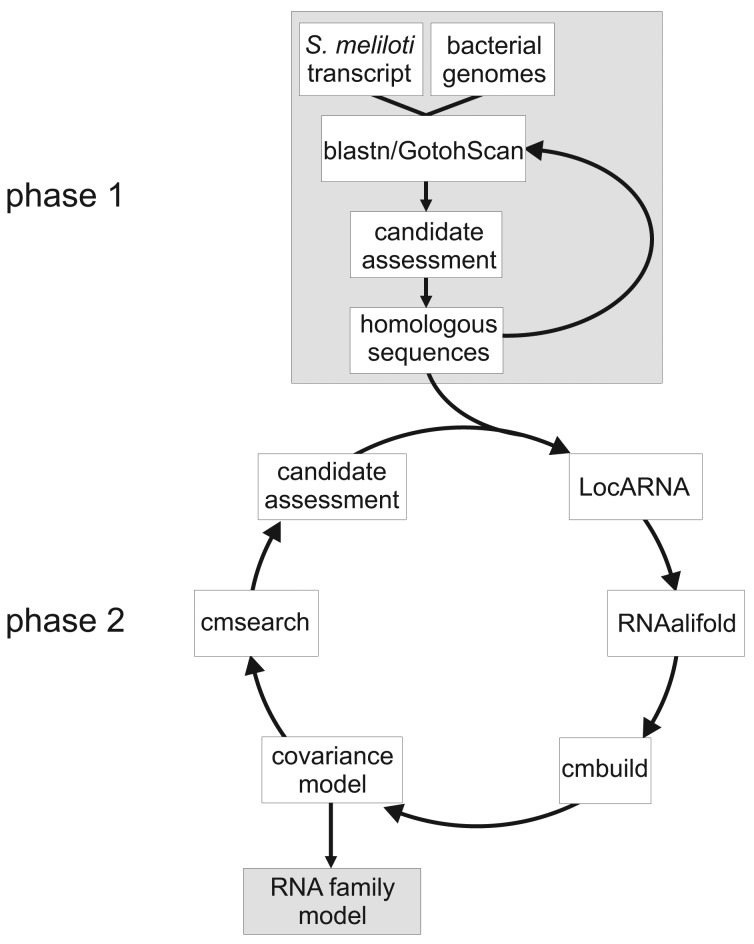
Workflow of covariance model construction.

Figure 1 gives an overview of our CM construction pipeline. Phase 1 identifies putative homologous RNAs by iterative searches focusing on sequence similarity. Phase 2 constructs an initial family model based on sequence and conserved structure, and uses this model to search all Rhizobiales for further homologs. After adding these to the family, Phase 2 is also iterated.

**Figure 2:**
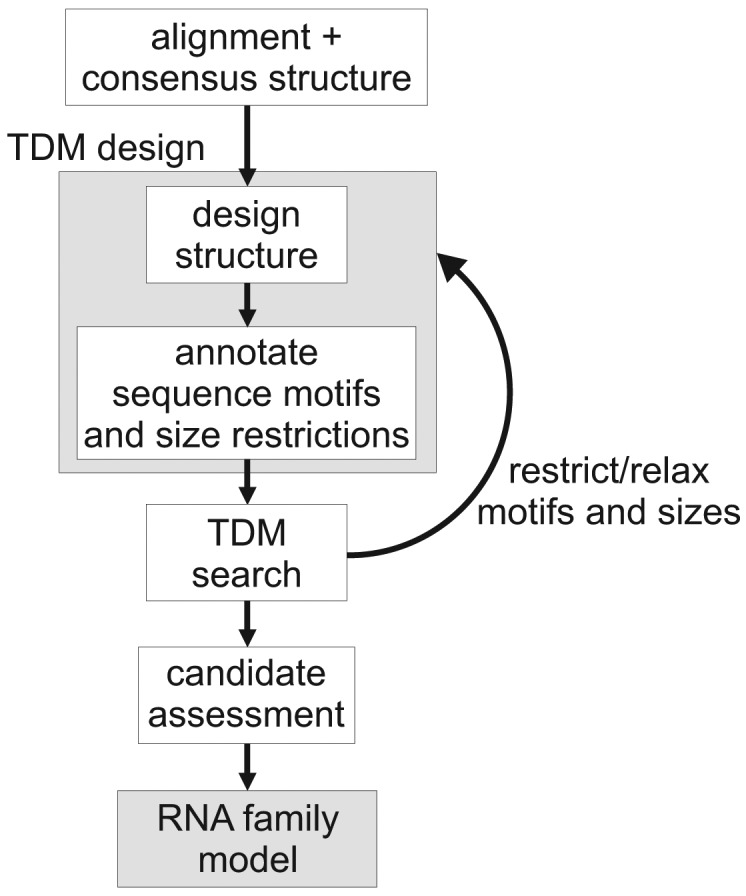
Workflow of thermodynamic matcher construction.

Figure 2 gives an overview of the TDM construction process. Here, we start from a transcript that has a well-defined secondary structure. First, we create a graphical description of this structure, using the Locomotif editor. The graphics can be annotated with size constraints for structural components, and with required sequence motifs. The graphics is then compiled by locomotif into a TDM. We use the TDM to scan other bacterial genomes in order to find subsequences that fold well into the described structure motif. The assessment of candidates is used to adapt the design of the TDM to be more restrictive or more relaxed.

Both methods of family model construction use the same assessment step, which checks for further evidence: Preservation of synteny, quality of alignment against the pivotal transcript, energy of a free folding. For the details, we refer the reader to the Methods section.

The family models are named after their pivotal elements, e.g., *RFM_SmelA_*_001_. These RFMs by themselves constitute an essential part of the results of this study, as they can be used (and extended further) to increase our knowledge about sRNAs in bacteria—beyond the findings that are reported here.

### 2.2. Distribution Pattern of Trans-Encoded sRNAs in the Rhizobiales

**Figure 3:**
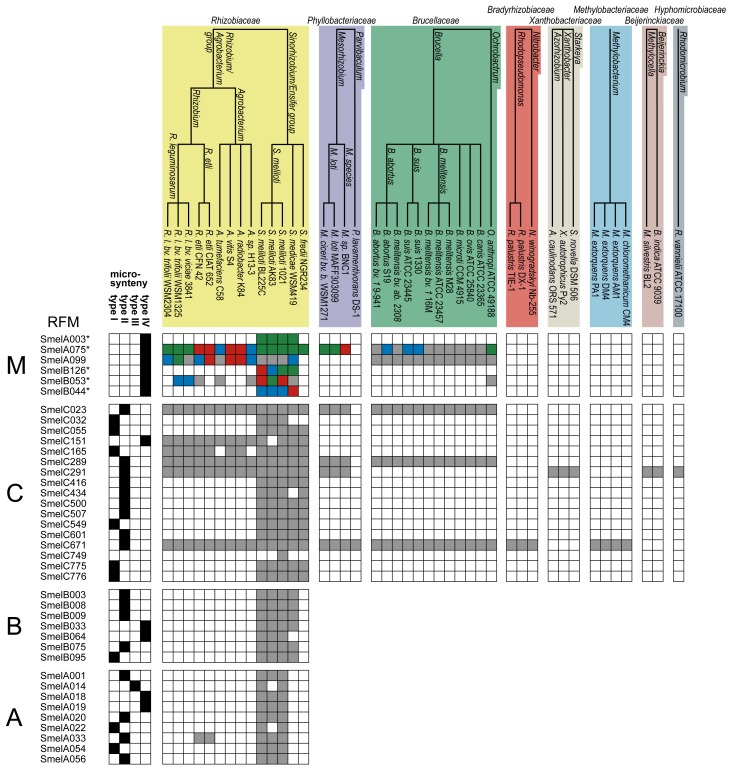
Distribution pattern of trans-encoded sRNAs in the Rhizobiales. The simplified phylogenetic tree includes sequenced strains and was adopted from the Pathosystems Resource Integration center (PATRIC) [48]. Analyzed bacterial strains that reveal no relatives in each RFM were removed from this scheme. A complete summary of all genomes used in this study is given in Table S2. sRNA occurrence of particular RFMs is given in each line. Chromosome (**C**), pSymA (**A**), and pSymB (**B**) of *S. meliloti* 1021 carry the initial set of trans-encoded sRNAs used in this comparative study and were indicated as different blocks separated by black, horizontal lines. The upper block (**M**) summarizes RFMs of sRNAs with several gene copies in particular genomes. * indicates RFMs that contain several sRNA gene copies in the *S. meliloti* 1021 strain (Table S1). The color code indicates the number of related sRNAs in each strain: 1 = grey, 2 = blue, 3 = red, and ≥4 = green. Complete (type I), extensive (type II), partial (type III), and fragmented (type IV) microsynteny is represented by black boxes.

The 52 analyzed trans-encoded sRNAs from *S. meliloti* and their relatives are collected in 39 RFMs (Figure 3, Table S1). At the first glance, they show a distribution of sRNAs in good accordance with phylogeny. In this subsection, we study their distribution in detail, moving from *S. meliloti* species to higher taxonomic levels. We will refer to Figure 3 and Table S1 throughout this discussion.

Among our 52 transcripts, 34 map to a single origin in *S. meliloti* 1021 and give rise to 34 RFMs. These 34 transcripts reveal a relative distribution of 50% (17), 29.4% (10) and 20.6% (7) on the chromosome, pSymA and pSymB, respectively. The remaining 18 transcripts reveal strong sequence similarity to other transcripts, originate from multiple loci and replicons, and were summarized to five RFMs *RFM_SmelA_*_003_, *RFM_SmelA_*_075_, *RFM_SmelB_*_044_, *RFM_SmelB_*_053_, and *RFM_SmelB_*_126_.

#### 2.2.1. Trans-Encoded sRNAs Delimited to the *S. meliloti* Strains 1021, BL225C, and AK83

Eleven of our transcripts appear to be restricted to *S. meliloti* strains, which share a core genome of approximately 5,100 genes dispersed on three replicons, a single chromosome, a second chromosome/megaplasmid and a symbiotic megaplasmid, respectively [40–42,49]. However, in AK83 two additional small plasmids were identified with a few genetic features corresponding to syntenic regions of the 1021 and BL225C symbiotic replicons [49]. RFMs of SmelA001, SmelA018, SmelA019, SmelA020, SmelA054, SmelA056, SmelB064, and SmelC032 reveal homologous sequences in the *S. meliloti* strains 1021, BL225C and AK83, while relatives of SmelA014 and SmelA022 are limited to BL225C, the most closely related strain of *S. meliloti* 1021 [49]. No homologous sequences were identified in case of SmelC749. Thus, it represents the only trans-encoded sRNA specific for *S. meliloti* 1021 identified in our study. RFMs deduced from pSymA-located sRNAs are composed of relatives located on the replicons psiNMEB01 and chromosome 3 of *S. meliloti* BL225C and AK83, respectively. Both, psiNMEB01 of *S. meliloti* BL225C and chromosome 3 of AK83 share functional similarities with the symbiotic megaplasmid pSymA. Along this line, the RFM members of SmelB064 (pSymB) are located on pSymB-like replicons (BL225C psiNMEB02 and AK83 chromosome 2), while SmelC032 relatives are located on chromosomal-like replicons (BL225C chromosome and AK83 chromosome 1) [40,41,49].

The RFMs of SmelA001, SmelA014, SmelA018, SmelA019, SmelA020, SmelA022, SmelA054, SmelA056, SmelB064, and SmelC032 contain relatives delimited to the three *S. meliloti* strains, which predominantly map to their particular symbiotic plasmids. Consequentially, on an evolutionary scale the emergence of these transcripts is a more recent *S. meliloti*-specific incident rather than a loss of the sRNA during evolution of all non-*S. meliloti* strains. This is in good agreement with Galibert *et al*. [40], who concluded that pSymA was acquired more recently in the evolutionary history of *S. meliloti*. Genomic analyses revealed a divergence of genome contents between pSymA and the two remaining replicons [40]. González *et al.* [50] hypothesized that emergence, remodeling, and annihilation of accessory plasmids in general, is highly variable within the Rhizobiales [50,51]. Our findings support the conclusion that emergence of trans-encoded sRNAs on accessory plasmids in general and on *S. meliloti*-specific symbiotic plasmids in particular is predominantly a recent evolutionary event. However, SmelB044 and SmelB064 have emerged on the pSymB-like replicon while SmelC032 and SmelC749 evolved on the ancestral chromosome. Thus, *S. meliloti*-specific sRNA emergence is not restricted to the symbiotic plasmids.

#### 2.2.2. Trans-Encoded sRNAs in the Genus *Sinorhizobium*

18 RFMs show an extended set of relatives in the *Sinorhizobium/Ensifer* group. The genus, among others, is composed of the most closely related bacteria *S. meliloti* 1021, BL225C, and AK83, the next related *S. medicae* WSM419, and *S. fredii* NGR234 with the biggest phylogenetic gap to *S. meliloti*.

Trans-encoded sRNAs of *RFM_SmelA_*_003_, *RFM_SmelB_*_003_, *RFM_SmelB_*_008_, *RFM_SmelB_*_009_, *RFM_SmelB_*_033_, *RFM_SmelB_*_044_, *RFM_SmelB_*_075_, *RFM_SmelB_*_095_, and *RFM_SmelB_*_126_ were identified in *S. medicae* but not in *S. fredii*. The *S. medicae* WSM419 genome consists of four replicons, a circular chromosome and three plasmids, pSMED01 (1.5 Mbp), pSMED02 (1.2 Mbp), and pSMED03 (0.2 Mbp) [52]. The genomic distribution of these sRNAs reveals a strong overrepresentation (89%) on pSymB-like replicons (which are represented by pSMED01 in *S. medicae*).

In contrast, *RFM_SmelC_*_055_, *RFM_SmelC_*_416_, *RFM_SmelC_*_434_, *RFM_SmelC_*_500_, *RFM_SmelC_*_507_, *RFM_SmelC_*_549_, *RFM_SmelC_*_601_, *RFM_SmelC_*_775_, and *RFM_SmelC_*_776_ commonly include additional relatives in both *S. medicae* and *S. fredii. S. fredii* NGR234 has a single chromosome (3.93 Mbp) and two additional plasmids, pNGR234a (0.54 Mbp) and pNGR234b (2.43 Mbp), whereof the smaller plasmid encodes the symbiotic features [51]. RFMs derived from trans-encoding sRNAs occurring in all *Sinorhizobium* strains, including *S. fredii*, are composed of members that predominantly map to the chromosomal replicons. This is a notable difference to the 20 RFMs which are restricted to *S. meliloti* and *S. medicae* strains and whose members predominantly map to the megaplasmids. An exception is given by a single relative of SmelC434 that is located on megaplasmid pNGR234b in *S. fredii*. With exception of the multicopy sRNAs SmelA003 and SmelB126, not in a single case unique sRNAs were found on the symbiotic plasmids. This is in good agreement with the strong fluctuation of accessory plasmids [53]. Furthermore, sizes of the symbiotic plasmids in *S. fredii* (0.54 Mbp) and *S. meliloti* strains (1.3 Mbp) differ by approximately 0.8 Mbp [41,51] and thus indicate a broad remodeling pattern even in the closely related members of the *Sinorhizobium/Ensifer* group.

#### 2.2.3. Trans-Encoded sRNAs in the *Rhizobiaceae*

Most of the analyzed sRNA families (32 out of 39) are restricted to the *Rhizobiaceae*. RFMs of SmelA033, SmelC151, and SmelC165 hold members with origins in the *Sinorhizobium*, *Rhizobium*, and *Agrobacterium* species. *RFM_SmelA_*_033_ shows an unusual distribution pattern with representatives in *S. meliloti*, *S. medicae*, and *R. etli. RFM_SmelC_*_151_ comprises members distributed in the whole *Rhizobiaceae* except for the *Candidatus liberibacter* genus and *S. meliloti* AK83. *RFM_SmelC_*_165_ displays a similar pattern but lacks relatives in *A. tumefaciens* and *A. radiobacter*.

#### 2.2.4. Complex Distribution of Trans-Encoded sRNAs in the Order of Rhizobiales

Slater *et al.* [53] proposed that the ancestor of the Rhizobiales is an unichromosomal organism that acquired an additional, ancestral plasmid [53]. This was underlined by a high proportion of conserved, primary chromosomes in Rhizobiales genera, e.g., *Rhizobium*, *Sinorhizobium*, *Brucella*, *Bradyrhizobium*, and *Mesorhizobium*, respectively. The theory of an ancestral plasmid was supported by the existence of several gene clusters that are conserved on the second chromosomes/megaplasmids while generally missing on the primary chromosomes [53]. Further, due to intragenomic gene transfers essential genes, e.g., the tRNA-Arg encoding gene in *S. meliloti*, are sporadically rearranged to the second chromosomes/megaplasmids [40,53]. Further replicons, in addition to the ancestral chromosome and plasmid, were determined as accessory plasmids with beneficial but non-essential features [53]. All this is in good agreement with our findings about sRNAs in the Rhizobales.

The comprehensive RFMs of SmelA075, SmelA099, SmelB053, SmelC023, SmelC289, SmelC291, and SmelC671 comprise members in the *Phyllobacteriaceae*, *Brucellaceae*, *Bartonellaceae*, *Bradyrhizobiaceae*, *Methylobacteriaceae*, *Beijerinckaceae*, and *Hyphomicrobiaceae*. The occurrence of these transcripts is restricted to the chromosomal replicons with an exception of *RFM_SmelA_*_075_, *RFM_SmelA_*_099_, and *RFM_SmelB_*_053_, whose members occur several times in each genome with copies on each replicon. RFMs of SmelC023 and SmelC289 show an equal distribution pattern in the Rhizobiales. Both are represented by 29 sRNA relatives in the *Rhizobiaceae*, *Brucellaceae*, and *Phyllobacteriaceae*. SmelC671 has additional relatives in *Bradyrhizobiaceae* and *Methylobacteriaceae. RFM_SmelC_*_291_ exhibits a more fragmentary occurrence in the Rhizobiales with relatives in the *Rhizobiaceae*, *Phyllobacteriaceae*, *Xanthobacteriaceae*, *Beijerinckaceae*, and *Hyphomicrobiaceae*.

RFMs of SmelA075, SmelC023, SmelC289, SmelC291, and SmelC671 show a broad distribution pattern within the Rhizobiales. Each sRNA family has relatives on primary chromosomes in the Rhizobiales strains and thus an ancestral trans-encoded sRNA for each of these models presumably arose in the beginning of the Rhizobiales evolution. However, *RFM_SmelA_*_075_ consists of presumably paralogous copies on different replicons, e.g., each replicon in *S. meliloti* 1021 harbors at least a single copy. The strong conservation in the Rhizobiales and the occurrence of at least a subset of copies on primary chromosomes suggest that the duplication and transfer events have initially been emanated from origins on ancestral chromosomes. Members of *RFM_SmelC_*_291_ and *RFM_SmelC_*_671_ indeed are distributed to the whole Rhizobiales, but are differentially lacking in several taxonomy families, e.g., *Bartonellaceae*, *Bradyrhizobiaceae* and *Xanthobacteriaceae*.

Similar to that, the *Rhizobiaceae*-specific *RFM_SmelA_*_033_ and *RFM_SmelC_*_165_ show a dispersed occurrence pattern, the former with representatives only in the *R. etli* strains. The latter is widely distributed in the *Rhizobiaceae* but does not occur in *A. tumefaciens* C58 and *A. sp.* H13-3. Presumably, the functional relevance of these transcripts has been lost since the specific emergence of these taxonomy families in their ecological niche. Generally, trans-encoded sRNAs act via base pairing with their mRNA targets or interact with RNA binding proteins [6]. In a precedent evolutionary step the target mRNA was presumably removed from the genome or somehow disrupted. This event in turn left a redundant, non-functional sRNA that was removed in the course of time. *RFM_SmelC_*_151_ has relatives in the *Rhizobium/Agrobacterium* as well as the *Sinorhizobium/Ensifer* group and serves as a good example for a *Rhizobia*-specifc sRNA.

Our study reveals relatives of SmelA033, SmelA075, SmelA099, SmelB053, SmelC023, SmelC151, SmelC165, SmelC289, SmelC291, and SmelC671 in both *Rhizobium etli* species. A genome wide tiling array study for *R. etli* CFN42 [30] identified, among others, 17 noncoding RNAs. ReC06, ReC25, ReC26, and ReC71 were re-identified within this study as relatives of SmelC023, SmelC289, SmelC291, and SmelC671, respectively. Related transcripts of *RFM_SmelC_*_291_ were confirmed via Northern blot analyses in *S. meliloti*, *S. fredii*, *R. etli* and *R. leguminosarum* strains and thus underlines the informative value of the comparative approach applied in this study [54]. Recently, a deep sequencing study using the 454-pyrosequencing technology identified about 228 noncoding transcripts located on the three *A. tumefaciens* C58 replicons. A subset of 22 sRNAs were additionally confirmed via Northern blot analyses. Eight of the RFMs computed in our study comprise members in *A. tumefaciens* and five, namely C1 (RNA8 of *RFM_SmelA_*_075_), C2 (RNA12 of *RFM_SmelC_*_023_), C5 (RNA11 of *RFM_SmelC_*_289_), C6 (RNA12 of *RFM_SmelC_*_291_), and L5 (RNA25 of *RFM_SmelA_*_099_) were experimentally verified in *A. tumefaciens* by both deep sequencing and Northern blot experiments [31].

Remarkably, each RFM with relatives beyond the *Sinorhizobium/Ensifer* group has no corresponding sRNA in the genus *Liberibacter*. Phylogenetic analyses identified the *Liberibacter* species as the most divergent species within the *Rhizobiaceae* with the largest distance to the root of this family. Thus, it might explain the lack of homologs of *Rhizobiaceae*-specific sRNAs in this genus [55]. Even for the most conserved models, e.g., *RFM_SmelC_*_671_, relatives in the *Liberibacter* genus were not found. In this context, it has to be noted that the genome of the *Liberibacter* genus consists of a relatively small chromosome, only 1.2 Mbp in size. Compared to the remaining *Rhizobiaceae*, enormous genomic capacity, including open reading frames and sRNA genes, has been lost within the *Liberibacter* lineage.

### 2.3. Microsynteny

Microsynteny means the preservation of the adjacent protein-coding gene upstream or downstream of a putative sRNA locus. Gene function is usually not affected by its location in relation to its genomic neighborhood. Consequentially, the degree of synteny is lost much faster than sequence similarity and represents a sensitive indicator for genome evolution [56–58].

To classify the dimension of microsynteny for the 39 RFMs, four categories were specified.

- Complete microsynteny (type I) is determined for relatives of both neighboring genes;
- extensive microsynteny (type II) means the majority of genes shares homology but with a few exceptions;
- partial microsynteny (type III) specifies the homology to a single adjacent gene and
- fragmented microsynteny (type IV) is given by subsets of homologous genes within a RFM.

According to this definition, microsynteny of type I, II, III, IV was observed for 9, 17, 1, and 11 RFMs, respectively (Figure 3, Table S1). Microsynteny analyses for the stand-alone sRNA SmelC749 was not performed.

Complete microsynteny was observed for RFMs SmelA022, SmelA054, SmelB095, SmelC032, SmelC055, SmelC165, SmelC549, SmelC775, and SmelC776, which are predominantly (8 out of 9) restricted to the *Sinorhizobium/Ensifer* group. An exception is given by *RFM_SmelC_*_165_ with relatives in the *Rhizobiaceae*. Extensive microsynteny was observed for RFMs of SmelA001, SmelA020, SmelA056, SmelB003, SmelB008, SmelB009, SmelB075, SmelC289, SmelC416, SmelC500, SmelC507, SmelC601, SmelC671, SmelC023, SmelC434, SmelA033, and SmelC291 (Figure 3, Table S1). Similar to the aforementioned RFMs, with the exception of SmelA033, SmelC023, SmelC289, SmelC291, and SmelC671, this type of microsynteny was predominantly observed in the *Sinorhizobium/Ensifer* group as well. As expected, the degree of microsynteny is higher for RFMs that are restricted to closely related organisms and to RFMs with a predominant occurrence on descendants of the ancestral chromosome and megaplasmid [53,56].

All RNAs of *RFM_SmelC_*_023_ are located adjacent to a DNA polymerase I encoding gene, except for RNA12 of *A. tumefaciens* str. C58. Furthermore, for the 14 RNAs identified in the *Rhizobiaceae*, a gene encoding a MarR-type transcriptional regulator is situated next to and in case of RNA4 of *S. fredii* overlaps the sRNA gene (Figure 5c, Table S1). Due to the aberrant length of the overlapping transcriptional regulator gene (compared to its homologous genes) and the presence of alternative start codons approximately 200 nt downstream of the predicted start we presume an annotation mistake. Except for RNA22, RNA25, and RNA28 of *B. abortus* S19, *B. ovis* ATCC25840 and *B. melitensis* M28, all *RFM_SmelC_*_023_ members that occur in the *Brucellaceae* are located antisense to a predicted small peptide encoding region. Due to the fact that the sequence of the predicted ORFs is also present in other *Brucellaceae* which lack this annotation, most likely this ORF was missed during gene prediction.

Except for RNA3 of *S. medicae* WSM419, all relatives of SmelC289 are located next to a prolyl-tRNA synthetase gene (Figure 6c, Table S1). In case of RNA11, RNA13, RNA14, RNA15, RNA17, RNA19, RNA20, and RNA21 of the *Brucellaceae*, the prolyl-tRNA synthetase gene is indeed located adjacent to the corresponding sRNA genes, but these sRNA genes are overlapped in antisense by one or two presumably misannotated, small hypothetical genes. For 18 RFMs, overlapping genes were predicted of which the majority is annotated as hypothetical and thus their function and existence remain in question.

Partial microsynteny was only observed for *RFM_SmelA_*_014_, while fragmented microsynteny is given for RFMs of SmelA003, SmelA018, SmelA019, SmelA075, SmelA099, SmelB033, SmelB044, SmelB053, SmelB064, SmelB126, and SmelC151 (Figure 3, Table S1).

**Figure 4:**
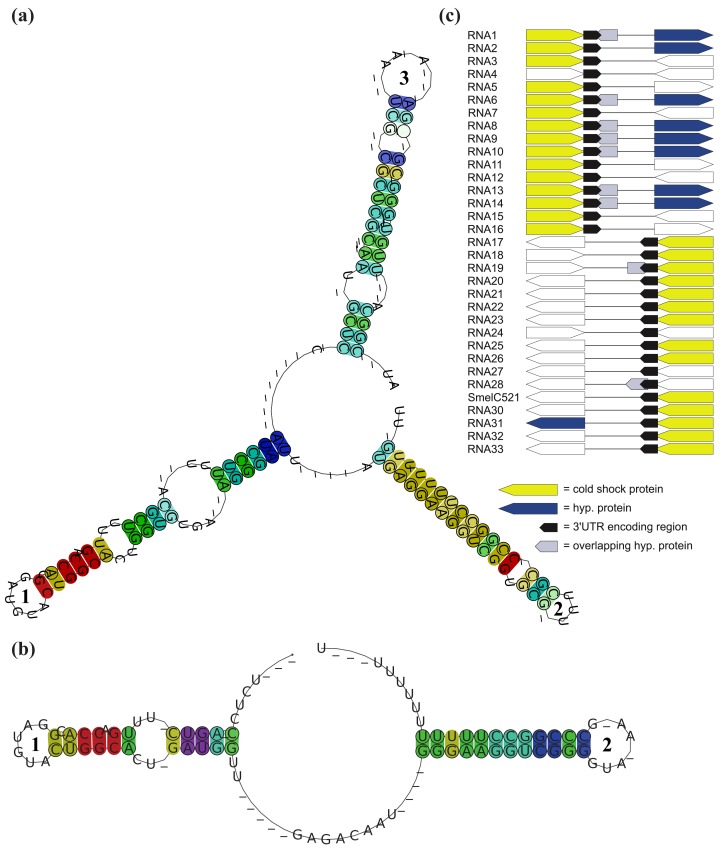
Structural comparison between *RFM_SmelB_*_053_ and related 3′-UTRs. (**a**) Consensus secondary structure of *RFM_SmelB_*_053_ and (**b**) related 3′-UTRs; Base pairs in (**a**) and (**b**) are colored according to the Vienna RNA conservation coloring scheme [65]. Colors indicate the number of nucleotide combinations, out of the six possible base pairs, in the underlying alignment that are involved in forming predicted base-pairs (red = 1, yellow = 2, green = 3, cyan = 4, blue = 5, purple = 6). Pale colors are used for the case that some sRNAs do not form a base-pair; (**c**) Associated proteins of 3′-UTRs. Arrows indicate the orientation of each gene, identical colors indicate homologous genes. Non-colored arrows denote non-homologous genes (genes are not shown to scale).

**Figure 5:**
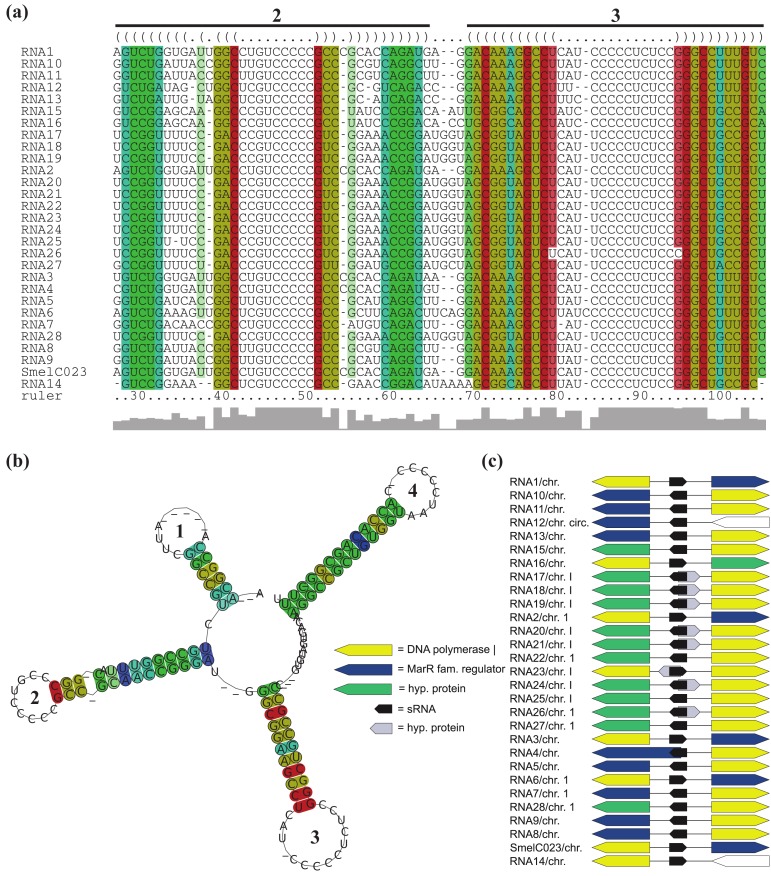
Structural, functional and genomic features of *RFM_SmelC_*_023_. (**a**) Alignment of presumed functional hairpin loops; and (**b**) consensus secondary structure of identified relatives of SmelC023. Base pairs are colored as in Figure 4a; (**c**) Microsynteny pattern of *RFM_SmelC_*_023_. Illustration as in Figure 4c.

In case of RFMs of SmelA003, SmelA075, SmelB053, SmelB044, and SmelB126, fragmented microsynteny is explained by their multiple copy numbers per genome. Members of these RFMs indicate a higher rate of intragenomic transfers. *RFM_SmelA_*_075_ and *RFM_SmelA_*_099_ occur with three and four hairpin loops, respectively, with similar loop motifs (see Section 2.7). Considering both RFMs, fragmented microsynteny is the dominant observation, but a closer look at specific taxonomy families, e.g., *R. leguminosarum* reveals “local” microsynteny. In detail, the *R. leguminosarum* relatives RNA9, RNA14, RNA16, RNA17, RNA18, RNA21, and RNA24 of *RFM_SmelA_*_099_ and RNA12, RNA17, RNA19, RNA26, RNA35, RNA42, and RNA54 of *RFM_SmelA_*_075_ occur in complete and extensive microsynteny, respectively *RFM_SmelB_*_126_ contains 4, 3, 2, and 5 copies in *S. meliloti* 1021, BL225c, Ak83, and *S. medicae* WSM419, respectively An association to a potassium transporter encoding gene was observed for at least a single sRNA copy in each genome. Thus it is tempting to speculate that the potassium transporter associated sRNAs represent the ancestral version of this RFM.

### 2.4. Copy Numbers and Association with Mobile Genetic Elements

Multiple copy numbers per genome as well as the scarce microsynteny of RFMs of SmelA003, SmelA075, SmelB044, SmelB053, and SmelB126 are in good agreement with the scattered occurrence of mobile genetic elements next to the sRNA loci. Mobile genetic elements probably contribute significantly to the genetic polymorphism in *S. meliloti* natural populations [59], since mobile genetic elements are able to copy and uncouple sRNA loci from their genomic context. Repeats and mobile genetic elements were also associated to members of *RFM_SmelA_*_014_, *RFM_SmelA_*_054_*, RFM_SmelB_*_003_, *RFM_SmelB_*_008_, *RFM_SmelB_*_009_, *RFM_SmelB_*_064_, *RFM_SmelB_*_075_, and *RFM_SmelC_*_500_.

### 2.5. Structural Features Conserved in RFMs

**Figure 6:**
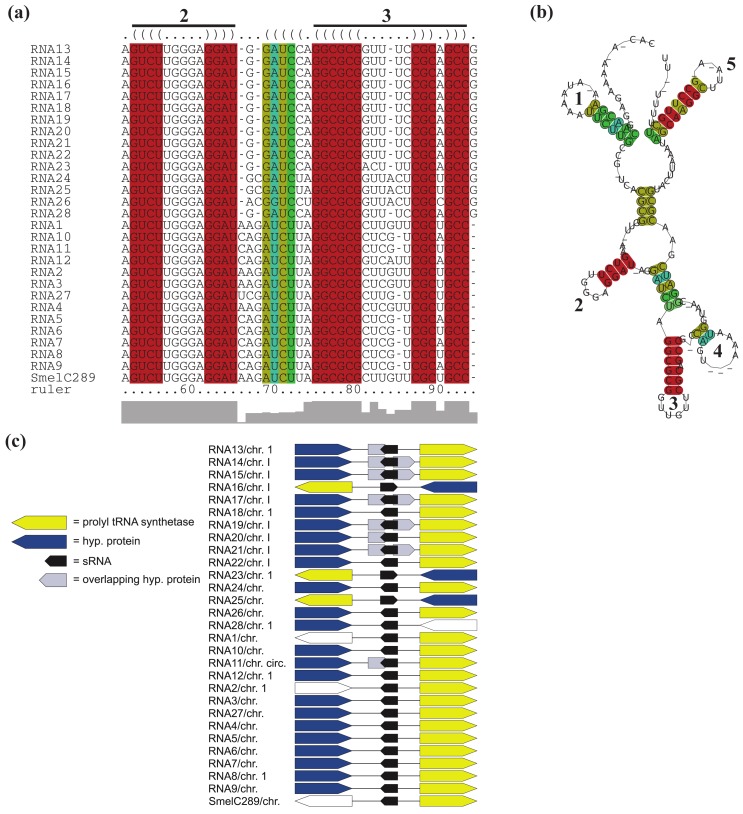
Structural, functional and genomic features of *RFM_SmelC_*_289_. (**a**) Alignment of presumed functional hairpin loops and (**b**) consensus secondary structure of identified relatives of SmelC289. Base pairs are colored as in Figure 4a; (**c**) Microsynteny pattern of *RFM_SmelC_*_289_. Illustration as in Figure 4c.

Generally, transcripts have a varying number of sub-structural RNA-domains determined as stacked base pairs, internal loops, bulges and hairpin loops [60]. A number of sRNAs, e.g., Yfr1 of several cyanobacteria, reveal typical Rho-independent terminator-like features with a 3′-located, GC rich hairpin followed by a poly-U-tail [61,62]. Additional examples for sRNAs with typical terminator features are represented by RprA and Qrr1 of *E. coli* and *Vibrio cholera*, respectively [63]. RFMs of SmelB126, SmelB053, SmelC434, SmelC507, SmelC151, SmelC023, SmelC289, and SmelC671 include typical terminator structures. On the contrary, *RFM_SmelB_*_075_, *RFM_SmelA_*_014_, *RFM_SmelA_*_054_, *RFM_SmelC_*_416_, *RFM_SmelC_*_601_, and *RFM_SmelC_*_165_ contain stems with atypical hairpin loops, e.g., disrupted with internal loops, followed by poly-U-tails (Figure 5b and Figure 6b, Supplement S1). Otaka *et al.* [64] reported that besides transcription termination, terminator poly-U-tails of the noncoding transcripts SgrS and RyhB in *E. coli* are essential for Hfq interaction and riboregulation [64]. A similar pattern could be presumed in case of the aforementioned trans-encoded sRNA models. However, the remaining models reveal no terminator-like features (Supplement S1).

A complex situation is given for SmelB050, SmelB053, and SmelC691. The pSymB-located sRNA genes share typical trans-encoded sRNA gene features with a long distance to neighboring genes. Their transcripts have distinct 5′- and 3′-ends and form a triple stem loop structure [38]. SmelC691 has a similar pattern except for the first stem loop. The identified sRNA relatives, all collected in *RFM_SmelB_*_053_, are mainly found in the *Rhizobiaceae*; only a single member occurs in *Ochrobactrum anthropii* in the *Brucellaceae* (Figure 3, Table S1). Comparison of all homologous sequences indicates the first stem loop as the most variable, sometimes completely missing domain, while the second stem loop shows strong conservation, at least in the loop motifs (GGAUGUA). The third stem loop has typical Rho-independent terminator-like features (Figure 4a, Supplement S1). In addition to the identified sRNA relatives of *RFM_SmelB_*_053_, a number of putative 3′-UTRs were identified in the Rhizobiales, which occur in a sequence and structure pattern similar to the second and third stem loop of the SmelB053 relatives (Figure 4b). The majority of the identified 3′-UTRs (29 out of 33) are connected to genes coding for proteins involved in cold shock adaptation, e.g., SmelC521 in *S. meliloti* 1021 (Figure 4c, Table S2) [38]. Post-transcriptional regulation of cold shock genes via special 3′-UTR structures was reported in case of the 428 nt long *cspA* mRNA in *E. coli*. The mRNA has two stem loop structures at its 3′-end connected to regulation of degradation via binding of Hfq and Poly-(A)-polymerase I (PAP I) that prevents binding of polynucleotide phosphorylase and RNAseE [66,67]. Due to the structural similarity of the homologous sequences identified in our study compared to the *cspA* 3′-UTR of *E. coli* and the predominant connection of the homologous 3′-UTRs to cold shock genes in the Rhizobiales, we suggest similar functions of the 3′-UTRs in a Hfq dependent manner. The functional characteristics of the trans-encoded sRNA SmelB053 and its relatives remain unclear, but due to their conspicuous similarity to the conserved 3′-domains of cold shock genes, they might act as interceptor transcripts that sequester RNA degradation complexes and thus protect and stabilize mRNAs. Sequestration is a well characterized phenomenon in bacteria, e.g., exemplified by CsrB, 6S and GlmY RNA [14,63,68].

Strong sequence and thus structure conservation is a general feature between RFM members of *S. meliloti*-specific sRNAs (SmelA001, SmelA014, SmelA018, SmelA019, SmelA020, SmelA022, SmelA054, SmelA056, SmelB064, SmelC032). The lowest conservation is found in *RFM_SmelB_*_064_ with a structure conservation index (SCI, see Methods section) of 0.91, while the remaining transcripts of each model reveal strong sequence and thus structure conservation, with SCI values of approximately 1 (Supplement S1). Functional characteristics of trans-encoded sRNAs are commonly provided by sub-sequences and structures, e.g., in case of DsrA and RyhB in *E.coli* [63]. Consequently in sRNA relatives, these crucial domains of sRNAs should be conserved in a more stringent manner than in non-functional domains. However, due to the kinship between and the high sequence similarities of the *S. meliloti* sRNAs, conclusions about conspicuous functional sub-structures of these transcripts remain impractical. As a matter of course, we see more sequence divergence for a subset of 22 RFMs with additional relatives in the *Rhizobiaceae. RFM_SmelA_*_003_ and *RFM_SmelB_*_126_ have several genome internal copies in each strain (Figure 3, Table S1). Presumably due to the duplication events, transcripts of these RFMs show strong sequence divergence. However, the structure as well as a motif section of the second hairpin loop of *RFM_SmelA_*_003_ shows conservation (Supplement S1). *RFM_SmelB_*_126_ reveals piecewise sequence conservations at more or less conserved positions, as well as a conserved 3′-located terminator hairpin. The best structural conservation for the 22 RFMs is represented by *RFM_SmelC_*_055_ (SCI = 1.04), *RFM_SmelC_*_151_ (SCI = 1.02), *RFM_SmelB_*_009_ (SCI = 1.02), *RFM_SmelC_*_500_ (SCI = 1.01), *RFM_SmelC_*_775_ (SCI = 1.0), *RFM_SmelB_*_075_ (SCI = 1.0), and *RFM_SmelB_*_008_ (SCI = 1.0) (Supplement S1). The strongest nucleotide divergence is shown by *RFM_SmelC_*_549_ with relatives in *S. meliloti* and *S. fredii* (SCI = 0.69) (Supplement S1).

In general, *RFM_SmelC_*_549_ consists of four conserved stem loops but is disrupted by several internal loops and single stranded domains. These single stranded domains are the most deviating regions and thus they are responsible for the depressed SCI value. This high conservation implies a functional role of these four domains (Supplement S1).

The RFMs of SmelC023 (SCI = 1.11), SmelA075 (SCI = 0.99), SmelC289 (SCI = 0.9), SmelC291 (SCI = 0.55), and SmelC671 (SCI = 0.55) are composed of transcripts with variable sequence and structure conservations, presumably derived from a common ancestor (Supplement S1). In case of *RFM_SmelC_*_023_ and *RFM_SmelC_*_289_, four and five hairpin loops, respectively, are the main components of these transcripts. The 5′-located domain of *RFM_SmelC_*_023_ is highly variable both in length and nucleotide composition. The 3′-located stem loop of *RFM_SmelC_*_023_ is a Rho-independent terminator-like structure with high GC content (Supplement S1). This is supported by the poly-T-sequence following the annotated sRNA gene (data not shown). Presumably a degradation event of the initially annotated sRNA SmelC023 occurred and resulted in a processed 3′-end. The middle domain consists of two hairpin loop structures with highly conserved sequences within the loops, while the structural maintenance of these sub-domains is provided by stems with varying nucleotide compositions with evolutionary established base pair exchanges (Figure 5a,b, Supplement S1). This strongly suggests that the functional maintenance of this molecule is provided by both the hairpin structures and the loop sequences. sRNAs with several functional domains are a common feature in bacteria. Obvious examples are the trans-encoded sRNAs OxyS and DsrA in *E. coli*. The former binds to the fhlA mRNA in a Hfq-dependent manner. Two stem loops are presumed to be involved in mRNA binding, while the interaction site of Hfq is different to that of the mRNA binding site [3,69,70]. The latter exhibits different hairpins for different targets. In detail, DsrA consists of three stem loops of which the second is able to interact with *hns* mRNA, blocks the ribosome binding site (RBS) and thus inhibits mRNA translation. The third stem loop interacts with the *rpoS* mRNA and activates translation via remodeling of an inhibitory mRNA sub-structure [70]. Similar features could be presumed for *RFM_SmelC_*_291_ (sra33) [54]. The 5′-domain of *RFM_SmelC_*_291_ has a structurally conserved stem loop with a strongly conserved loop motif, UCCGCCGCAUCU, while the second stem loop shows extremely variable stem sequences but occurs with a dominant but different loop motif, UCCUCG as well (Supplement S1).

*RFM_SmelC_*_289_ shows a 5′-located stem loop characterized by an AU-rich loop, stabilized by a variable stem of organism-specific nucleotide contents. Similar to *RFM_SmelC_*_023_, the 3′-region contains a Rho-independent terminator-like structure and the middle domain consists of two conserved hairpin loops as well. The former hairpin shows complete conservation, while the latter reveals a highly conserved stem with a variable loop (Figure 6a,b, Supplement S1). However, the functional patterns of noncoding transcripts are not restricted to their presumed single stranded domains, e.g., the binding sites of RprA sRNA in *E. coli* are predominantly incorporated in stem structures [63]. Further hidden sRNA sequences that are essential for target interactions could be released due to a structural reformation of the sRNA, for example, the RNA binding protein Hfq is able to alter the secondary structure of the RydC sRNA in *Enterobacteriaceae* resulting in an active version of this transcript [71]. *RFM_SmelC_*_671_ represents the most variable model of related sRNAs in this study. The transcript has a long single-stranded domain with a conserved CUCCCUGU motif, enclosed by down- and upstream located, highly variable hairpin loops of which the 3′-located domain acts as terminator (Supplement S1). A similar pattern was observed for Qrr1 in *Vibrio cholera*. This transcript reveals a long single stranded domain between its first and second loop, which was verified as the mRNA interaction site. This motif is highly conserved within Qrr2-4, which are paralogs of Qrr1 [4,63].

### 2.6. sRNAs in Antisense

Large numbers of cis-encoded antisense RNAs were identified, e.g., via sequencing and tiling array studies in *Synechocystis* [28], *H. pylori* [26], *S. meliloti* [38], and *R. etli* [30]. Cis-encoded antisense sRNAs are located in antisense to their targets, act via perfect base pairing and mediate post-transcriptional regulation, e.g., stabilization or destabilization of target mRNAs [6]. Pairs of small noncoding transcripts that are located in antisense to each other remain rarely identified in bacteria. Georg and Hess [72] presumed that two small transcripts encoded by unlinked sRNA genes interact with each other due to a complementary section in both transcripts. However, the functional relevance of this feature needs to be further elucidated.

Here, we observe that SmelC776 relatives are located in antisense to *RFM_SmelC_*_775_ sRNAs and thus most likely allow a mutual interference of both transcripts. This is in good agreement with the strong sequence conservation of SmelC776 (SCI = 0.95) within the overlapping domain (Table S1, Supplement S1). Approximately 90% (17 out of 19) of the nucleotide exchanges of *RFM_SmelC_*_776_ in the *S. fredii* strain occur outside the overlapping part and thus suggest the antisense part as the functional transcript domain.

### 2.7. Focus on SmelA075 and SmelA099

**Figure 7:**
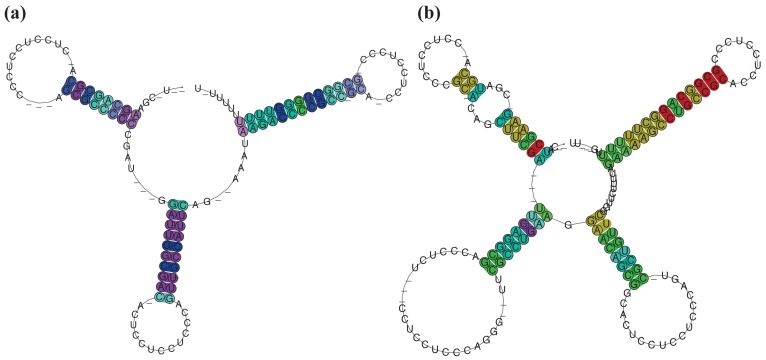
Hairpin loop structures of *RFM_SmelA_*_075_ and *RFM_SmelA_*_099_. (**a**) Consensus secondary structure of *RFM_SmelA_*_075_; and (**b**) *RFM_SmelA_*_099_. Base pairs are colored as in Figure 4a.

A remarkable situation was found in case of *RFM_SmelA_*_075_ and *RFM_SmelA_*_099_, which exhibit three and four hairpin loops, respectively. Each hairpin loop carries similar loop motifs, CCUCCUCCC, representing an anti Shine-Dalgarno (aSD) sequence, while the stems show more variability in their nucleotide content (Figure 7, Supplement S1). In several sRNAs, e.g., RNA III in *Staphylococcus aureus*, CyaR in *Enterobacteria* and ABcR1 in *A. tumefaciens* C58, loop sequences with at least partial aSD motifs were observed. Functional analyses demonstrated that the aSD motif is indispensable for sRNA binding to the RBS of the particular mRNA targets, as well as the resulting translation inhibition [73–79]. The sequencing profile as well as Northern blot analyses of SmelA075, which is a member of *RFM_SmelA_*_075_, suggested SmelA075 as a stress-induced sRNA that occurs in several processed forms [38]. This is in good agreement with the trans-encoded sRNA RS0680a and its homologous transcripts identified in *Rhodobacter sphaeroides*. RS0680a represents a shorter version than the RFM members identified in this study. The transcript has two stem loops instead of three and four, and each comprises an aSD motif. It was suggested that RS0680a undergoes different maturation processes and is involved in the stress response in a more general pattern via binding to the RBS of several genes [80]. From a biological point of view, it was suggested to group all derivatives of *RFM_SmelA_*_075_ and *RFM_SmelA_*_099_ to a single sRNA family that consists of members with a varying number of hairpin modules. Implementing all facts, this RNA family consists of several copies per genome. It is somehow involved in stress adaption, presumably in post-transcriptional regulation via blocking the RBS of target mRNAs.

## 3. Experimental Section

In this section, we describe in detail the process of RNA family model construction from our *S. meliloti* transcripts which is not a fully automated method. We report on the automatic steps and on the points where human judgement or design decisions are involved. A genuine knowledge of common bioinformatics tools is assumed. All tools used are strictly concerned with secondary structure—non-standard base pairs, possible pseudoknots, or other tertiary interactions are not considered. Although including such features would be desirable, present day tools cannot achieve this.

### 3.1. Sequence Data and Databases

52 of approximately 180 trans-encoded sRNAs were selected and downloaded from GenDB [81], accessible via the RhizoGATE portal [1,2]. The choice of these 52 candidates was made in purely technical terms: Candidate transcripts should be well-covered by sequence reads, with clearly defined ends, and remote from any coding region. We plan to generate models for the remaining transcripts in the near future.

Complete genome sequences and annotations of all *Rhizobiales* available were obtained from the NCBI genomes FTP site [82]. For a complete list see Table S3. Additionally, whole genome and plasmid sequences were included that are not (yet) part of the above collection. Sequence data of *A. sp. H13-3*, *B. melitensis M28*, *S. meliloti AK83*, and *S. meliloti BL225C*, all members of the order of the Rhizobiales, were downloaded from the NCBI nucleotide database.

### 3.2. Construction of RNA Family Models

There is no standard and fully automated way to construct an RNA family model. The general difficulty of this process has been discussed, e.g., in [83]. Family model construction is supported by a variety of tools, but interspersed with modeling decisions and candidate screening by a human expert. In our case, we start with a trans-encoded sRNA from *S. meliloti*, say SmelXnnn, and construct an RNA family model *RFM_SmelXnnn_*, which (1) comprises a set of orthologous RNA sequences from related organisms; and provides (2) a search function to find further family members in the Rhizobiales and beyond. In a few cases, we find that several sRNAs should be collected into the same family model, which is then named arbitrarily after one of them.

We constructed types of RFMs, *Covariance models* and *Thermodynamic matchers*. The use of these two complementary methods has already been motivated above (Section 2.1). We now add some details about both methods, and about the assessment step used with both.

### 3.3. Automated Candidate Generation and CM Construction Steps

Recall Figure 1, which gives an overview of our RFM construction pipeline. Phase 1 identifies putative homologous RNAs by iterative searches focusing on sequence homology. Phase 2 constructs an initial family model based on sequence and conserved structure, and uses this model to search all Rhizobiales for further homologs.

#### 3.3.1. Phase 1: Sequence Homology Search for SmelXnnn

In the first stage of the workflow, putative homologs are obtained by sequence homology searches using blastn [84] and GotohScan [85]. We initialize the search for homologous RNA sequences of a reference sequence SmelXnnn by employing blastn with *E* < 10^−5^ on the complete set of alphaproteobacterial genomes (For word-size and scoring function we set the parameters -W 7 -q -3 -r 2 -G 2 -E 2).

As fragmentation of conserved regions is a common characteristics of RNA families, candidate homologs often do not cover the complete reference sequence. Therefore, blastn matches must be postprocessed. First, the candidate sequence is extended on either side to cover the reference sRNA sequence plus an extra 10% of its length on either side. Next, the reference is semi-globally aligned to the candidate sequence and un-matched leading and trailing bases of the candidate are trimmed.

The detection of RNA homologs is complemented by GotohScan searches for SmelXnnn in three separated sequence databases representing the families of *Rhizobiaceae*, *Brucellaceae*, and *Phyllobacteriaceae*, using default parameters. The latter are the most closely related families of *Rhizobiaceae*, with *S. meliloti* as member, within the order of Rhizobiales.

Resulting candidates from both search methods are combined and undergo assessment in the same way.

#### 3.3.2. Iteration of Homology Search

As is common practice in search of distant homologies [86], rather than searching with a relaxed threshold, we use a stringent threshold in each step. From the hits determined and assessed positively, a new search emerges, again with a stringent threshold. We use a maximum of three iterations.

#### 3.3.3. Phase 2: CM Creation and Search

**Figure 8:**
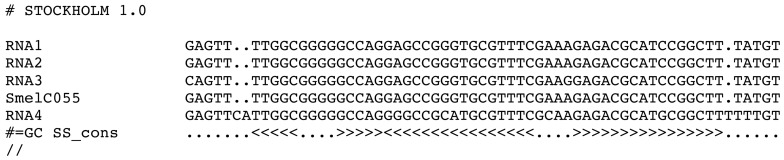
Multiple sequence alignment and consensus structure, as required for CM construction. Shown is the multiple sequence alignment of *RFM_SmelC_*_055_ in Stockholm format.

We enter Phase 2 with a first set of candidates for *RFM_SmelXnnn_*, given that at least two homologs of SmelXnnn were found. An initial covariance model *CM_SmelXnnn_* is to be built. This requires a multiple sequence alignment which supports a consensus structure. LocARNA [87] is used for creating the structural alignment, RNAalifold [88,89] for prediction of consensus structure from this alignment, and Infernal [24] for CM model construction and search. Figure 8 gives an example of the input for CM construction.

After constructing *CM_SmelXnnn_* by Infernal, a profile search is performed on all alphaproteobacterial genomes. The best 50 hits are analyzed and undergo assessment. The steps of model construction, search and hit assessment are repeated while new homologs are identified. A maximum of three cycles is allowed. The third iteration uses as a cut-off a CM-score of 25% of the highest CM-score of any present member of the model.

### 3.4. TDM Model Construction

For specific RNA families, we found that a CM was inadequate to express their peculiarities. For example, SmelA075 has three hairpins, all of which exhibit a perfect loop motif (CCUCCUCCC) (cf. Section 2.7). It is widely distributed among the Rhizobiales, with the stems sequences highly diverged. As a consequence, the discriminatory power of the CM decreases. A TDM can be designed such that it gives no weight to the stem sequences, but enforces the loop motif. SmelB053 is a similar case with a strongly conserved structure and low sequence similarity, except for a prominent loop motif (GAUGUA).

**Figure 9:**
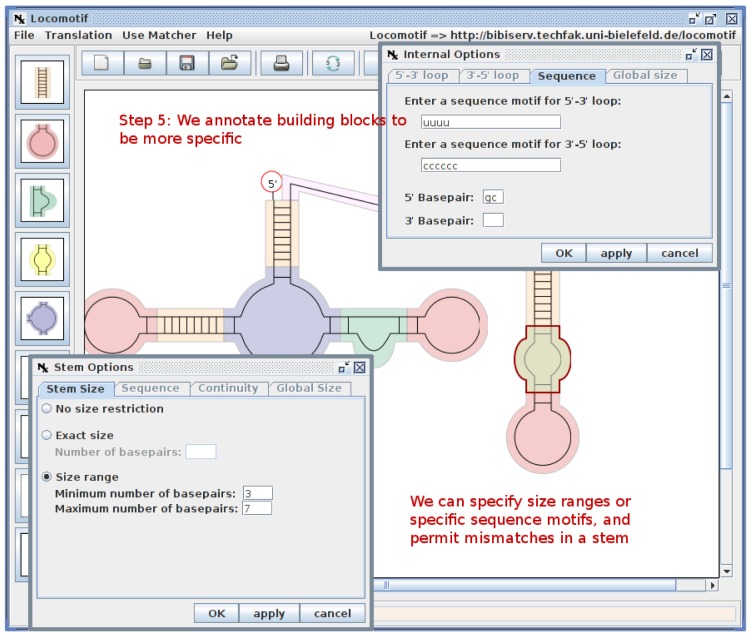
A snapshot from the construction of a thermodynamic matcher using the Locomotif editor.

Figure 9 shows a snapshot of TDM construction, where we depict a structure graphics with the help of the Locomotif editor [47], annotate it with conserved sequence information and compiled it into a search program at the Bielefeld Bioinformatics Server [90].

TDMs tend to run a bit faster than CMs (when their HMM filter is turned off). We use them to scan all *Rhizobiales* genomes. Resulting candidates are assessed like other candidates.

Design of a TDM also requires some iteration, as the motif description can be made more or less specific. Generally, it is a good strategy to begin with a rather restrictive motif and check that the known sRNAs are actually found, which verifies that the design is correct. Then, the motif is gradually relaxed. Search results may suggest adjustment to the original TDM design, such as relaxing the number of paired bases in a stem, or increasing the allowed size for a loop. This interplay of human design, matcher compilation and search continues as long as candidates pass the assessment step.

### 3.5. Assessment: Taming the Flood of Candidates

When an RNA family model is available, its search procedure can be used with more or less stringent cut-offs. In this study, however, we need to construct such a model in the first place. We start from a single family member, which may not even be a typical one, but has the virtue of being based on an experimental screen rather than being an *in silico* prediction. We want to collect a large number of homologs, and use further evidence to weed out unplausible candidates.

**Figure 10:**
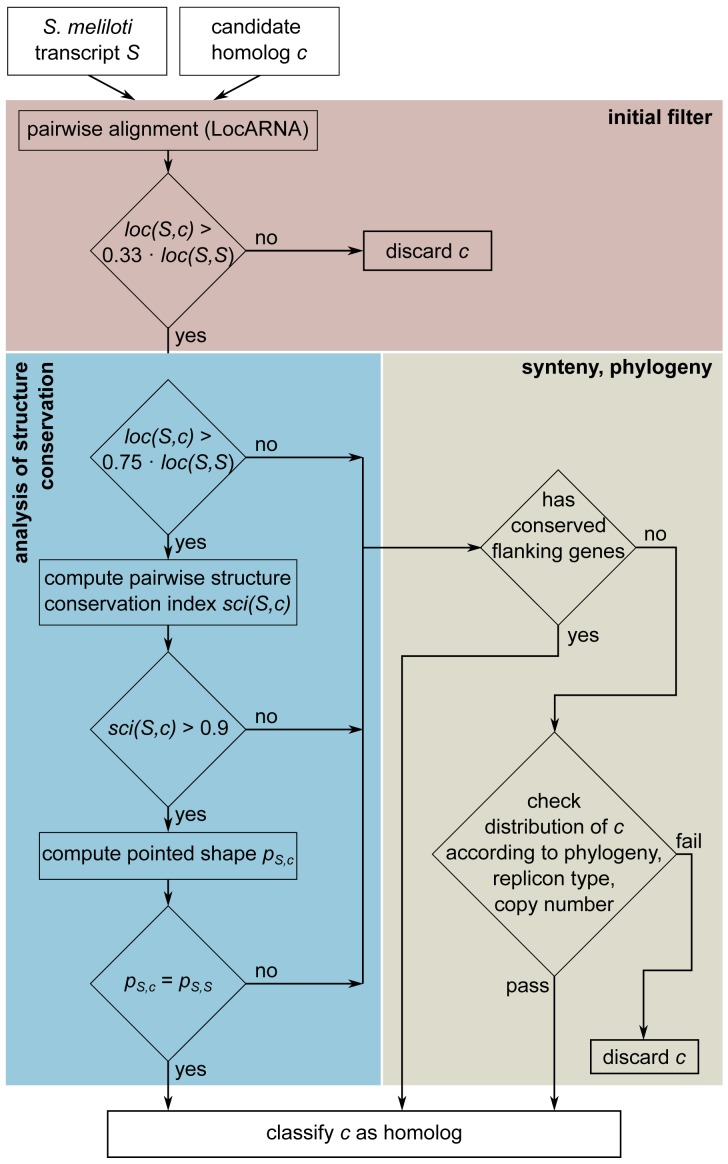
Candidate selection process.

Some sequences show strong sequence and structure conservation and thus can be unambiguously identified in an automatic fashion. No further effort, human or computational, is spent on them. Divergent sequences without global conservation have to be curated integrating sequence and structure conservation with additional sources of information, such as genomic context and phylogenetic distribution. Figure 10 gives an overview of this assessment, which is described further below.

#### 3.5.1. Filtering of Sequence Homlogs Based on Pairwise LocARNA Scores

Let *S* = SmelXnnn for this section. Each putative homolog returned by sequence search is aligned (separately) to the *S* using LocARNA. For any further consideration, only candidates *c* are retained for which their LocARNA score *loc*(*S*, *c*) satisfies

loc(S,c)>0.33⋅loc(S,S)

This restriction is due to practical considerations, as a trade off between the number of candidates that we have to inspect against the possible loss of true homologs. It leaves us with a set of candidates that align well to *S* individually, yet the structures underlying their alignments may have little in common.

#### 3.5.2. Analysis of Structure Conservation

We next promote candidates with strong structural conservation, which pass the selection process without further inspection: From the previous step, we have two criteria that measure individual structure conservation of each candidate *c* against S, the (pairwise) LocARNA score *loc*(*S*, *c*) and the (pairwise) structure conservation index *sci*(*S*, *c*), which is computed with the help of RNAfold and RNAalifold, both part of the Vienna RNA Package [91]. If an SCI value is low, the candidate rather folds into a native structure different from the consensus found by LocARNA. Now the pieces of information gained from pairwise considerations have to be related.

For the pairwise alignment of *S* and *c*, we map the consensus structure back to *S* and compute its abstract shape representation, computed with RNAshapes [92,93], extended by hairpin centers (akin to the “helix centers” of [94]). A hairpin center is calculated as (*i* + *j*)/2, where *i* and *j* are the positions of the hairpin closing base pair.

We refer to the combination of shape and hairpin center as a *pointed shape*. This yields (potentially different) pointed shapes *p_S_*_,_*_c_* for *S*, one for each candidate *c*. We also compute *p_S_*_,_*_S_*, which is the pointed shape of the minimum free energy structure for *S*. Candidate *c* qualifies as a family member if

(1) loc(S,c)>0.75⋅loc(S,S)and

(2) sci(S,c)>0.9and

(3) $$p_{S,c}=p_{S,S}$$

The last equation borrows the idea of consensus *shapes* from [95] for a fast way to select candidates where structure conservation is obvious.

Candidates which do not pass the above test are not (yet) discarded, but subjected to the next step.

#### 3.5.3. Synteny, Phylogeny, and Multiple Alignment

We perform BLAST comparisons of the protein-coding genes flanking SmelXnnn against the Rhizobiales database, with a maximum E-value of 10^−6^ to indicate gene synteny. If one or both flanking genes are conserved with respect to at least one of the family members as accepted at this point, the candidate is accepted.

Additionally, we examine the distribution of homologs related to phylogeny, replicon type, and copy number. Candidates are discarded, for example, when located on a different replicon in a closely related strain, or when there is only a single hit in a remote phylogenetic group.

Finally (this must be the last assessment step for computational reasons), candidates that passed the previous criteria are cast into a multiple structural alignment with LocARNA. Such an alignment is easily derailed by outliers that do not fit to a common structure. Obvious outliers, exhibiting a scattered alignment throughout the sequence, are removed by human inspection. As long as candidates are removed, the alignment is recalculated for the remaining family members.

### 3.6. Towards a More Automated RNA Family Model Construction Process

So far we completed 39 RFMs including 52 of 173 trans-encoded sRNAs published in [38]. There are 121 more to go. Further automation of the model construction process is highly desirable, but not easy to achieve. While some parts of our assessment step can be integrated into an automated workflow, there are also serious challenges arising from technical limitations of the available software. We discuss two such aspects in the sequel.

#### 3.6.1. Modular Architecture of RNA Families

An important characteristic of RNA is its modular architecture. Similar substructures, shared among different RNA families, or multiple copies of modules within an RNA family may indicate related functionality. In RNA family reconstruction, shared modules complicate the identification of homologs by making it difficult to distinguish whether a candidate belongs to an already existing family or constitutes a completely new RNA family, merely sharing a similar sub-structure.

An example for different manifestations of a repetitive module, comprised of a single hairpin, are the trans-encoded sRNAs SmelC201 (not included in this study), SmelA075, and SmelA099. Their structures are composed of two, three, and four consecutive copies of similar hairpins. Neither CMs nor TDMs (at least not those created with Locomotif) can model a variable number of modules. In case of SmelA075 and SmelA099, our workaround was to build separate models for each module number. An extra classification step was required for matches obtained from the alternative models, because shorter models produced multiple overlapping hits to homologs that were members of RFMs with a higher number of module copies. From the algorithmic point of view, it should be simple to extend modeling techniques in this direction.

#### 3.6.2. Conserved Terminators in Short sRNAs

Finding homologs for sRNAs with a length below 80 nucleotides is generally difficult if their sequence has diverged while retaining a conserved structure. In particular, when the structure is a GC-rich hairpin, it often matches to terminator hairpins in multiple locations. A source of confusion is for example *RFM_SmelB_*_053_, whose structure consists of three adjacent hairpins. The nuceotide sequence of the central hairpin is conserved, whereas the last one constitutes a *bona fide* terminator.

Situations like this ask for a generalization of models towards avoidance of specified motifs. This is probably more difficult than allowing for optional modules.

## 4. Conclusions

In this study, we aimed at the identification of homologous sRNAs in the Rhizobiales, starting with a set of well-defined trans-encoded sRNAs from *S. meliloti* 1021. This is the first comprehensive, comparative *in silico* approach in this group of bacteria. Definition of RNA family models and grouping of sRNAs into these families is complicated by the poor knowledge about relationships between sequence, structure, and functions of sRNA domains. Whereas strong sequence and structural conservation is a good indication for assignment to the same family, the process becomes more difficult if only short sequence motifs and some structural features show similarities. This also includes ambiguous situations of sRNAs showing limited similarities to different family models. Therefore, full automation of RFM construction has not yet been achieved in this study. It is also not clear how far up in the taxonomy an approach like ours can reach. Hence, numbers of false positives and negatives are likely to increase with the evolutionary distance of the organisms.

Several independent pieces of evidence suggest that the 39 family models delivered here are a trustworthy bases for further, experimental and bioinformatics analyses. Apart from the criteria of sequence, structure and synteny conservation, such independent evidence is the following:

- Our initial, experimental screen [38] was able to recover transcripts of the majority of sRNAs known in *S. meliloti* at that time;
- Generally, our family models exhibit a plausible distribution of their members with respect to phylogeny;
- In particular, the specific distribution of observed transcripts on replicons is in agreement with the accepted view that the symbiotic plasmid is a late acquisition in *Sinorhizobium*;
- Members of five of our family models, found in *A. tumefaciens*, were validated experimentally by deep sequencing and Northern blots in independent studies (cf. Section 2.2).

Our approach provides valuable insights into the distributions of conserved and the presence of species-, family-, and genus-specific sRNAs. Most of the RNA families are restricted to the *Rhizobiaceae*, but a few show a broader distribution, implying a more general conserved function. While functional studies of sRNAs may build on our predictions, the future bioinformatics tasks are to construct models for the remaining transcripts from *S. meliloti*, and the extension of the comparative analysis to the alphaproteobacteria, and possibly beyond.

## Supplementary Files

Supplementary File 1 ZIP-Document (ZIP, 882 KB)
