# Supplementary material for: Conservation and Occurrence of Trans-Encoded sRNAs in the Rhizobiales
Source: Genes (Basel). 2011 Nov 18;2(4):925–56. doi: 10.3390/genes2040925 (PMC3927594; doi:10.3390/genes2040925)

All images in this document use the Vienna RNA conservation coloring scheme. Colors indicate the number of nucleotide combinations, out of the six possible base pairs, in the underlying alignment that are involved in forming predicted base-pairs (red = 1, yellow = 2, green = 3, cyan = 4, blue = 5, purple = 6). Pale colors are used for the case that some sRNAs do not form a base-pair.

RNA Family Model of SmelA001. (**a**) Alignment of $RFM_{SmelA001}$ sequences (SCI = 0.97); (**b**) Consensus secondary structure (minimum free energy -10.65 kcal/mol) for the alignment shown in (**a**).

(a)

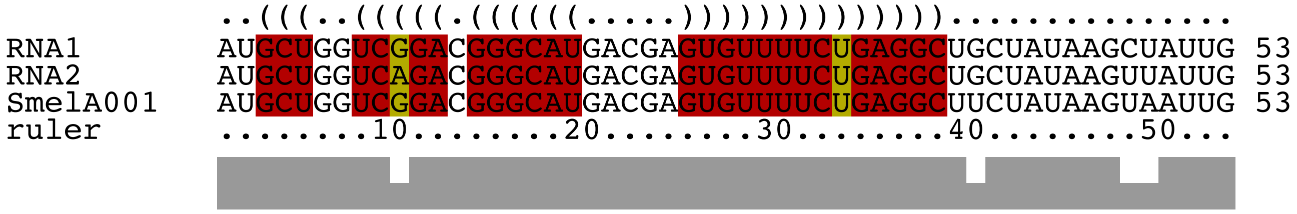

(b)

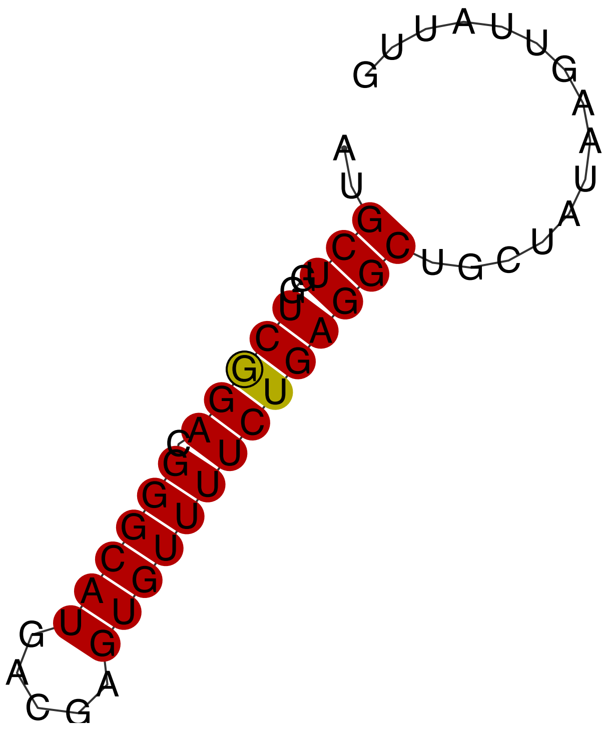

RNA Family Model of SmelA003. (**a**) Alignment of $RFM_{SmelA003}$ sequences (SCI = 0.73); (**b**) Consensus secondary structure (minimum free energy -32.39 kcal/mol) for the alignment shown in (**a**).

(a)

```
                    .........((((((...............))))))..............(((((((.(((((.(.....................))))))))))))
RNA1                UCA-CUUUCGAGCGG--CGAC--C--C-AGCCGCUUGG-GUGAGUGA-UGACCUCAC-AGCUG-UG-AAG-AAU-GC-CCCUUCUCCGCAGCUCUGAGGC   84
RNA10               UCA-CCC-CGAGCGGC-CGCCGUCG-CUCGCCCCUUGG-GUGAGUGA-UGGGCUUAG--GUUG-UA-AAA-A-UCGUCCCCUUCUCCACGGCUCUGAGGC   88
RNA11               UCA-CCC-CGAGCGGC-CGCCGUCG-UUAGUCCCUUGG-GUGAGUGA-UGGGCUUAG--GUUG-UA-AAA-A-UCGUCCCCUUCUCCACGGCUCUGAGGC   88
RNA12|SmelB046      UUA-CGUCCGAGCGUC-GGCC-UCGU--C---GCUCGAAGUGAGUGG-UGCCUCAGG-GCUGCAUA-GU----UCGU-CCCUUCU-GGGCAGCCCUGAGG   83
RNA13               UCA-CCCCCGAGCGGC-CCCC-UCGUC-AGCCGCUCGU-GUGAGUGAGUUCCUCAGC-GCUGC-CUUGCA---UUGU-CCCUUCU-AGGCAGCGCUGAGG   88
RNA14               UUA-CGUCCGAGCGUC-GGCC-UCGU--CGA-GCUCCAAGUGAGUGG-UGCCUCAGG-GCUGCAUA-GU----UCGU-CCCUUCU-GGGCAGCCCUGAGG   85
RNA15               UCAGC-CC-AAGCGGC-A--C-UCGUC-AGCCGCUCGG-GUGAGUGA-UGCCUCAGA-GCUGC-CC-GCA---UCGU-CCCUUCU-AGGAGGCUCUGAGG   83
RNA16               UCA-CCCCCGAGCGGCCCCCC-UCGUC-AGCCGCUCGA-GU--GUGG-UACCUCGGA-GCUGC-AU--AA---UUGU-CCCUUCA-AUGCAGCCCUGAGG   84
RNA17               UCA-CCCCCGAGCGGCCCCCC-UCGUC-AGCCGCUCGA-GU--GUGG-UACCUCGGA-GCUGC-AU--AA---UUGU-CCCUUCA-AUGCAGCCCUGAGG   84
RNA18               UCA-CCCCCGAGCGGCCCCCC-UCGUC-AGCCGCUCGA-GU--GUGG-UACCUCGGA-GCUGC-AU--AA---UUGU-CCCUUCA-AUGCAGCCCUGAGG   84
RNA19               UCA-U-GCCGGGCGGC-AACC-UCGUC-GGCCGCUCGU-GUGAGUGA-UGCCUCAGA-GCUGC-CC-GCG---UCGU-CCCUUCU-AGGAAGCUCUGAGG   85
RNA2                UCU-CGUCCGAGCGGCACCCC-UGG-C-GGCCGCUUGC-GUCAGUGA-UCGCCUCAG-AGCUG-CA-AAG-UUU-GU-CCCUUCGCCGCAGCUCUGAGGC   88
RNA20               UCA-C-CCGGAGCGGC-AUUC-UCGUC-UGCCGCUCGU-GUGAGUGA-UGCCUUAGA-GCUCC-CC-GCG---UCGU-CCCUUCU-GGGAAGCUCUGAGG   85
RNA21               UCA-C-CCGGAGCGGC-AUUC-UCGUC-UGCCGCUCGU-GUGAGUGA-UGCCUUAGA-GCUCC-CC-GCG---ACGU-CCCUUCU-GGGAAGCUCUGAGG   85
RNA22               UCA-U-GCCGAGCGGC-AACC-UCGUC-AGCCGUUCGU-GUGAGU-AGUGCCUCAGA-GCCGC-CG-GCG---CCGU-CCCUUCU-UGGCAGCUUUGAGG   85
RNA23               UCA-C-CCGGAGCGGC-AUUC-UCGUC-UGACGCUCGU-GUGAGUGA-UGCCUUAGA-GCUCC-CC-GCG---UCGU-CCCUUCU-GGGAAGCUCUGAGG   85
RNA24               UCA--CUCCGAGCGGC-ACCC-UCGUC-CGCCGCUCGG-GUGAGUGAAUGCCGCGGGACUGCC-CGAGC-----CGA-ACCUUC--CGGACAGCCCGAGG   85
RNA3                UCA-GUCCGGACGGC----AC-UCGUC-A-GCCGCUCG-GUGAAUGA-UGCCCUCAG-AGCUG-CACAAGCA---GU-CCCUUCUACGCAGCUUUGAGGC   84
RNA4                UCA-CCCUCGAGCGG--CGAC--C--C-AGCCGCUUGG-GUGAGUGA-UGGCCUCAG-AGCUG-UA-AAG-AUU-GU-CCCUUCUCCGCAGCUCUGAGGC   84
RNA5                UCA-CCCUCGAGCGG--CGAC--C--C-AGCCGCUUGG-GUGAGUGA-UGGCCUCAG-AGCUG-UA-AAG-AUU-GU-CCCUUCUCCGCAGCUCUGAGGC   84
RNA6                UCA-G-CCCGAGCGGC---AC-UCGUCAA-CCGCUCGG-GUGAGUGA-UGCCCUCAG-AGCUG-CACAAGUA---GU-CCCUUCUACGCAGCUUUGAGGC   85
RNA7|SmelC441       UCA-CCCUCGAGCGG--CGAC--C--C-AGCCGCUUGG-GUGAGUGA-UGGCCUCAG-AGCUG-UA-AAG-AUU-GU-CCCUUCUCCGCAGCUCUGAGGC   84
RNA8                UCA-CCC-CGAGCGGC-CGCCGUCG-CUAGCCCCUUGG-GUGAGUGA-UGGGCUUAG--AUUG-UA-AAG-A-UCGUCCCCUUCUCCACGGCUCUGAGGC   88
RNA9                UCA-GUCCGGGCGGC----AC-UCGUC-A-GCCGCUCG-GUGAAUGA-UGCCCUCAG-AGCUG-CACAAGCA---GU-CCCUUCUACGCAGCUUUGAGGC   84
SmelA003            UCA-CUUUCGAGCGG--CGAC--C--C-AGCCGCUUGG-GUGAGUGA-UGACCUCAG-AGCUG-UG-AAG-AAU-GC-CCCUUCUCCGCAGCUCUGAGGC   84
RNA25               ACG--ACCAAGGCGGC-ACCC-UCGUC-AGCCGCU-GCGGCGCCUGA--AGGUCCCG-GAGCC-AACGCG-A-UUGU-CCCUUCUAAACGCUCCGGGACC   87
RNA26               ACG--ACCACGGCGGC-AUCC-UCGUC-AGCCGUCCACGUCGUCUGA--AGGUCCCG-GAGCC-AACGCG-A-UUGU-CCCUUCUAAACGCUCCGGGACC   88
RNA27|SmelC144      ACG--ACCACGGCGGC-AUCC-UCGUC-AGCCGUCCACGUCGUCUGA--AGGUCCCG-GAGCC-AACGCG-A-UUGU-CCCUUCUAAACGCUCCGGGACC   88
RNA28               ACG--ACCACGGCGGC-AUCC-UCGUC-AGCCGUCCACGUCGUCUGA--AGGUCCCG-GAGCC-AACGCG-A-UUGU-CCCUUCUAAACGCUCCGGGACC   88
ruler               .........10........20........30........40........50........60........70........80........90.......100

                    .......
RNA1                U-CCAUU   90
RNA10               U-CCACC   94
RNA11               U-CCACC   94
RNA12|SmelB046      CAGUACC   90
RNA13               C-UCUCU   94
RNA14               CAGCACC   92
RNA15               C-GC-C-   87
RNA16               C-CCACC   90
RNA17               C-CCACC   90
RNA18               C-CCACC   90
RNA19               C-GC-C-   89
RNA2                U-UCACG   94
RNA20               U-GCGCA   91
RNA21               U-GCGCA   91
RNA22               C-GCACU   91
RNA23               U-GCGCA   91
RNA24               C-GCACU   91
RNA3                G-CCACC   90
RNA4                U-CCACC   90
RNA5                U-CCACC   90
RNA6                G-CCACC   91
RNA7|SmelC441       U-CCACC   90
RNA8                U-CCACC   94
RNA9                G-CCACC   90
SmelA003            U-CCACU   90
RNA25               U-UCC-U   92
RNA26               U-UCA-U   93
RNA27|SmelC144      U-UCA-U   93
RNA28               U-UCA-U   93
ruler               .......110
```

(b)

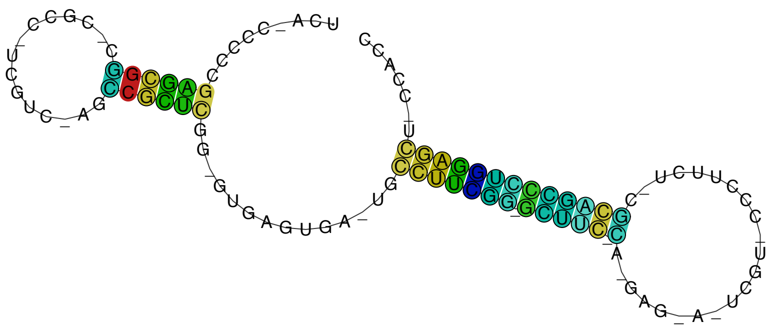

RNA Family Model of SmelA014. (**a**) Alignment of $RFM_{SmelA014}$ sequences (SCI = 1.01); (**b**) Consensus secondary structure (minimum free energy -54.90 kcal/mol) for the alignment shown in (**a**).

(a)

```
          .(((((((...((((((......((.((((((.....))))))))..))).)))...))))))).((((((((((((((.....)))..))))))))))))
RNA1      AUCACCCAAUUCAACGACUGUCUGCAGUGACGAUGAUCGUCGCGC-UUCGUUUGUCGUGGGUGAACGAGGGCGCUCCCCAUGACGGGUUGAGCGCCCUCG  99
SmelA014  AUCACCCAAUUCAACGACUGUCUGCAGUGACGAUGAUCGUCGCGCUUUCGCUUGUCGUGGGUGAACGAGGGCGCUCCCCAUGACGGGUUGAGCGCCCUCG 100
ruler     .........10........20........30........40........50........60........70........80........90.......100

          ....
RNA1      GCUU 103
SmelA014  GCUU 104
ruler     .......110
```

(b)

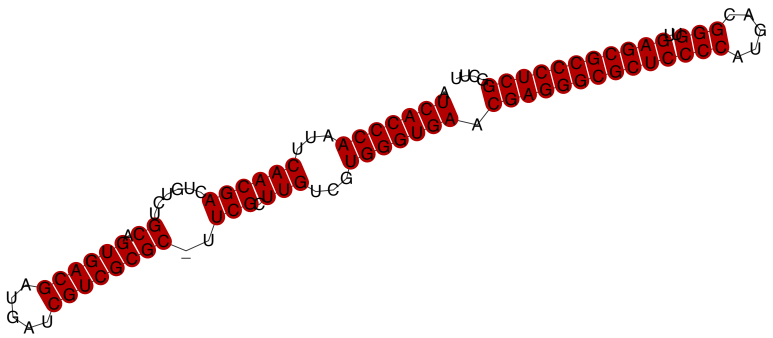

RNA Family Model of SmelA018. (**a**) Alignment of $RFM_{SmelA018}$ sequences (SCI = 1.00); (**b**) Consensus secondary structure (minimum free energy -25 kcal/mol) for the alignment shown in (**a**).

(a)

```
          ...............((.(((((((....(.....)..((((..(((((..((((...))))...))))).))))))).)))))).....
RNA1      ACGAUAAAACUUGAUAGUACCGGGCAUGACAGAAUGGACGGCAAGAGGAAACUGCUAUGCAGGGCUCCUCAGCCGGCUACGGUCUCACGC 90
SmelA018  ACGAUAAAACUUGAUAGUACCGGGCAUGACAGAAUGGACGGCAAGAGGAAACUGCUAUGCAGGGCUCCUCAGCCGGCUACGGUCUCACGC 90
RNA2      ACGAUAAAACUUGAUAGUACCGGGCAUGACAGAAUGGACGGCAAGAGGAAACUGCUAUGCAGGGCUCCUCAGCCGGCUACGGUCUCACGC 90
ruler     .........10........20........30........40........50........60........70........80........90
```

(b)

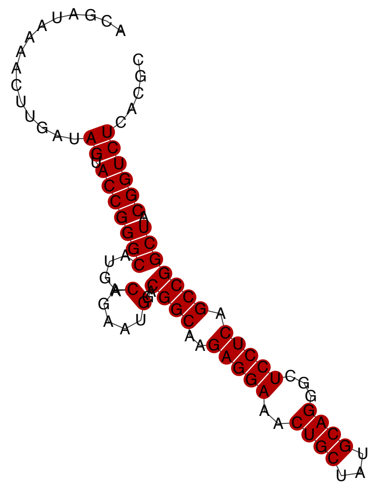

RNA Family Model of SmelA019. (**a**) Alignment of $RFM_{SmelA019}$ sequences (SCI = 0.98); (**b**) Consensus secondary structure (minimum free energy -32.80 kcal/mol) for the alignment shown in (**a**).

(a)

```
         ...((((...(((.(((((....))))))))...))))...((((.(((.....))).))))..((.(((((..........))))).)).......
RNA2     AUGGGAGAUUCGAUGCAGCGGGGGCUGCUCGUACCUCCACGGUCCAGCUGCUUAAGACGGACAACGGUGCUGAUCUGGUGCACAGCAACGGGAAUUC 97
SmelA019 AUGGGAGAUUCGAUGCAGCGGGGGCUGCUCGUACCUCCACGGUCCAUCUGCUUAAGACGGACAACGGUGCUGAUCUGGUGCACAGCAACGGGAAGUC 97
RNA1     AUGGGAGAUUCGAUGCAGC-GGGGCUGCUCGUACCUCCACGGUCCAUCUGCUUAAGACGGACAACGGUGCUGAUCUGGUGCACAGCAACGGGAAGUC 96
ruler    ........10........20........30........40........50........60........70........80........90.......
```

(b)

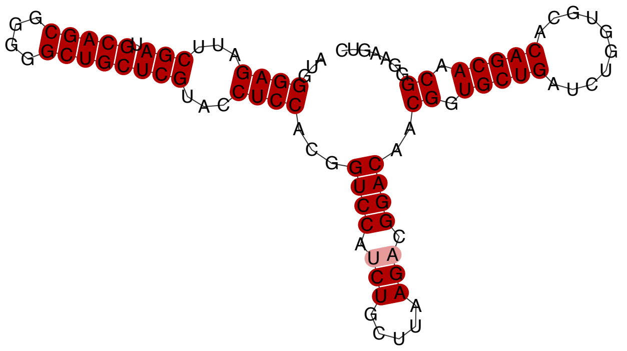

RNA Family Model of SmelA020. (**a**) Alignment of $RFM_{SmelA020}$ sequences (SCI = 1.00); (**b**) Consensus secondary structure (minimum free energy -44.50 kcal/mol) for the alignment shown in (**a**).

(a)

```
         ...((((......((((((((..((.((((((((....))))))))..))..)).)))))).))))).(((((((((((.(.((....).)..).)))))))
RNA1     ACUACCCGUAAUCAAUGACUACCUGCAGCGAUGGAACUCUGUCGCGCGUUCGUAAGUCGUCCGGGUCCGAAGGGCGCUCCCCGAAAGUGUCGUGCGCCCU 100
SmelA020 ACUACCCGUAAUCAAUGACUACCUGCAGCGAUGGAACUCUGUCGCGCGUUCGUAAGUCGUCCGGGUCCGAAGGGCGCUCCCCGAAAGUGUCGUGCGCCCU 100
RNA2     ACUACCCGUAAUCAAUGACUACCUGCAGCGAUGGAACUCUGUCGCGCGUUCGUAAGUCGUCCGGGUCCGAAGGGCGCUCCCCGAAAGUGUCGAGCGCCCU 100
ruler    ........10........20........30........40........50........60........70........80........90.......100

         )))..
RNA1     UCGUC 105
SmelA020 UCGUC 105
RNA2     UCGUC 105
ruler    .......110
```

(b)

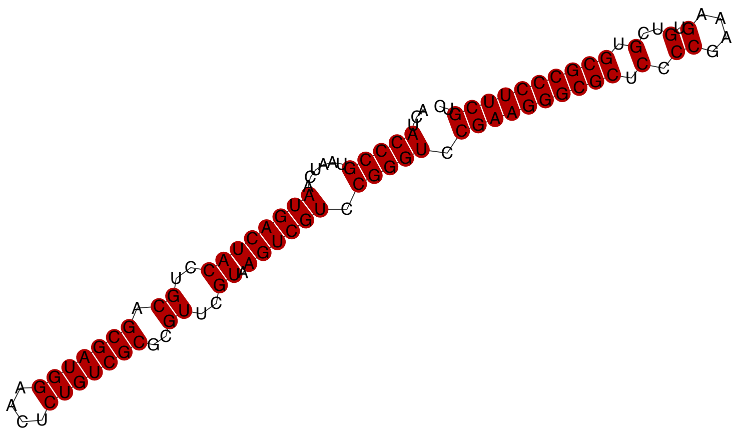

RNA Family Model of SmelA022. (**a**) Alignment of $RFM_{SmelA022}$ sequences (SCI = 1.00); (**b**) Consensus secondary structure (minimum free energy -56.20 kcal/mol) for the alignment shown in (**a**).

(a)

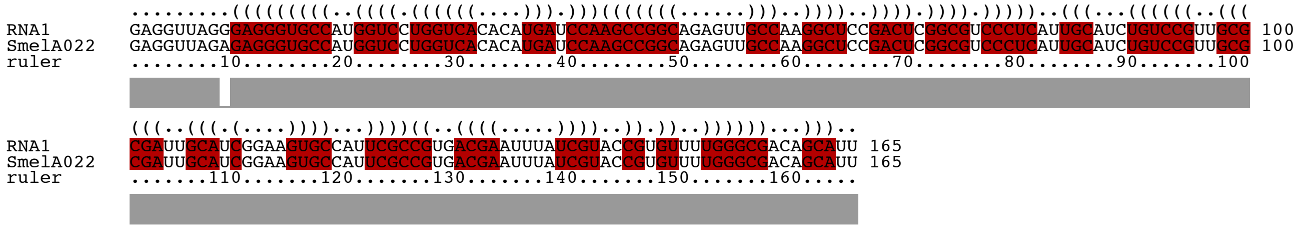

(b)

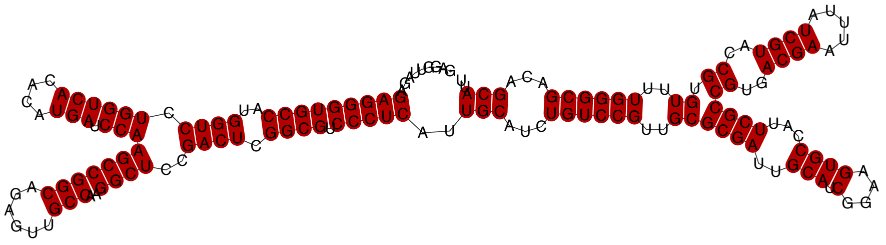

RNA Family Model of SmelA033. (**a**) Alignment of $RFM_{SmelA033}$ sequences (SCI = 0.92); (**b**) Consensus secondary structure (minimum free energy -31.88 kcal/mol) for the alignment shown in (**a**).

(a)

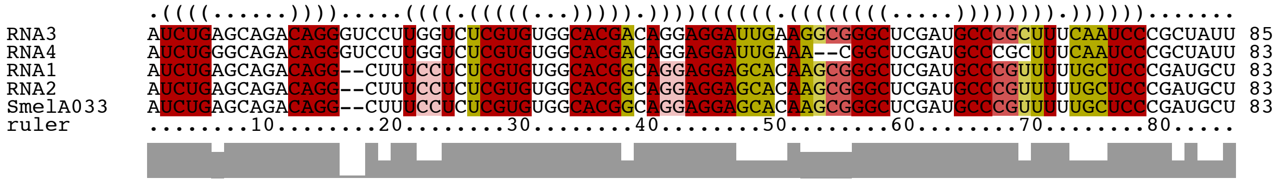

(b)

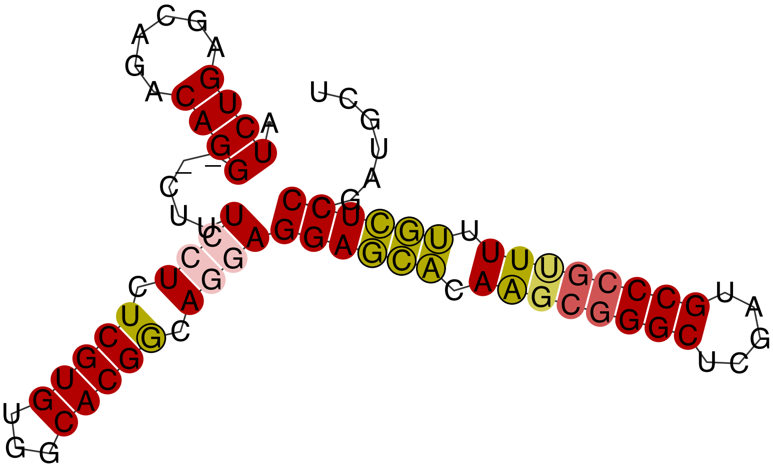

RNA Family Model of SmelA054. (**a**) Alignment of $RFM_{SmelA054}$ sequences (SCI = 1.00); (**b**) Consensus secondary structure (minimum free energy -25.40 kcal/mol) for the alignment shown in (**a**).

(a)

```
            ...........(((((((......))..((.(((((((((......((((((((((((.....)))))))))))))))))).))))).)))))......
RNA1        ACCCAUCAGAAGUAUUGAAGACGCUCAAGGAUGUGAUUGUUUUUUAGAGAGGAGUUCGCUCGACGGGCUCCUUUUGCGGUCAACACCAAAGUACCCCUUU 100
SmelA054    ACCCAUCAGAAGUAUUGAAGACGCUCAAGGAUGUGAUUGUUUUUUAGAGAGGAGUUCGCUCGACGGGCUCCUUUUGCGGUCAACACCAAAGUACCCCUUU 100
RNA2        ACCCAUCAGAAGUAUUGAAGACGCUCAAGGAUGUGAUUGUUUUUUAGAGAGGAGUUCGCUCGACGGGCUCCUUUUGCGGUCAACACCAAAGUACCCCUUU 100
ruler       .........10........20........30........40........50........60........70........80........90.......100
```

(b)

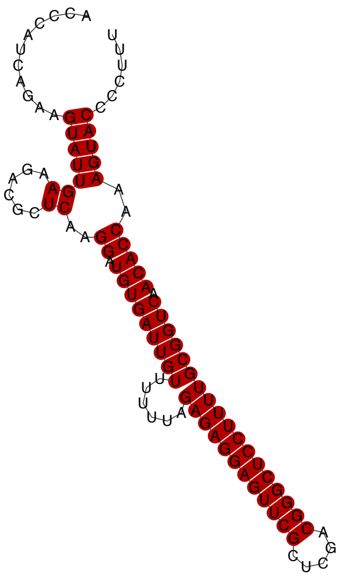

RNA Family Model of SmelA056. (**a**) Alignment of $RFM_{SmelA056}$ sequences (SCI = 1.00); (**b**) Consensus secondary structure (minimum free energy -74.30 kcal/mol) for the alignment shown in (**a**).

(a)

```
            ....(((((....)))))(((((.(((((.(((((...........)))))(((((((....))))))))))))).(((........)))..)))))....
RNA1        CUAUGGGAUCACUGUCCUGCUGCUGAUGAUCGAUCCUAACCGAAAAGAUCGCGCGCCGCGGACGGCGCGUCAUCCGCGAAUAUGCCCGCAUGCGGCAAUA 100
SmelA056    CUAUGGGAUCACUGUCCUGCUGCUGAUGAUCGAUCCUAACCGAAAAGAUCGCGCGCCGCGGACGGCGCGUCAUCCGCGAAUAUGCCCGCAUGCGGCAAUA 100
RNA2        CUAUGGGAUCACUGUCCUGCUGCUGAUGAUCGAUCCUAACCGAAAAGAUCGCGCGCCGCGGACGGCGCGUCAUCCGCGAAUAUGCCCGCAUGCGGCAAUA 100
ruler       .........10........20........30........40........50........60........70........80........90.......100

            ....((((((((...(((((((.........(((((........))))).((((((....))))))....))))))).)))))))).
RNA1        CGCACUCUGGCGAUAGGCGCGGAGACCGUCGGGUGGCCUCUGUCCCACUACGGUGAACGAUCACUGCUUACUGCGCCGCGUCAGGGG 187
SmelA056    CGCACUCUGGCGAUAGGCGCGGAGACCGUCGGGUGGCCUCUGUCCCACUACGGUGAACGAUCACUGCUUACUGCGCCGCGUCAGGGG 187
RNA2        CGCACUCUGGCGAUAGGCGCGGAGACCGUCGGGUGGCCUCUGUCCCACUACGGUGAACGAUCACUGCUUACUGCGCCGCGUCAGGGG 187
ruler       .......110.......120.......130.......140.......150.......160.......170.......180.......
```

(b)

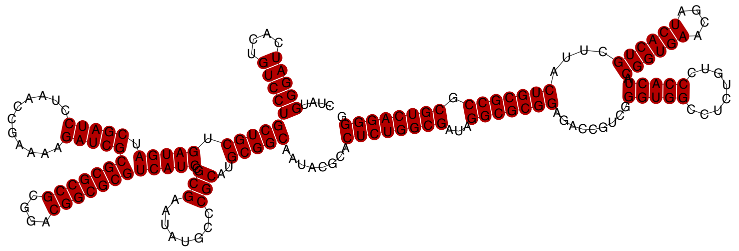

RNA Family Model of SmelA075. (**a**) Alignment of $RFM_{SmelA075}$ sequences (SCI = 0.99); (**b**) Consensus secondary structure (minimum free energy -52.38 kcal/mol) for the alignment shown in (**a**).

(a)

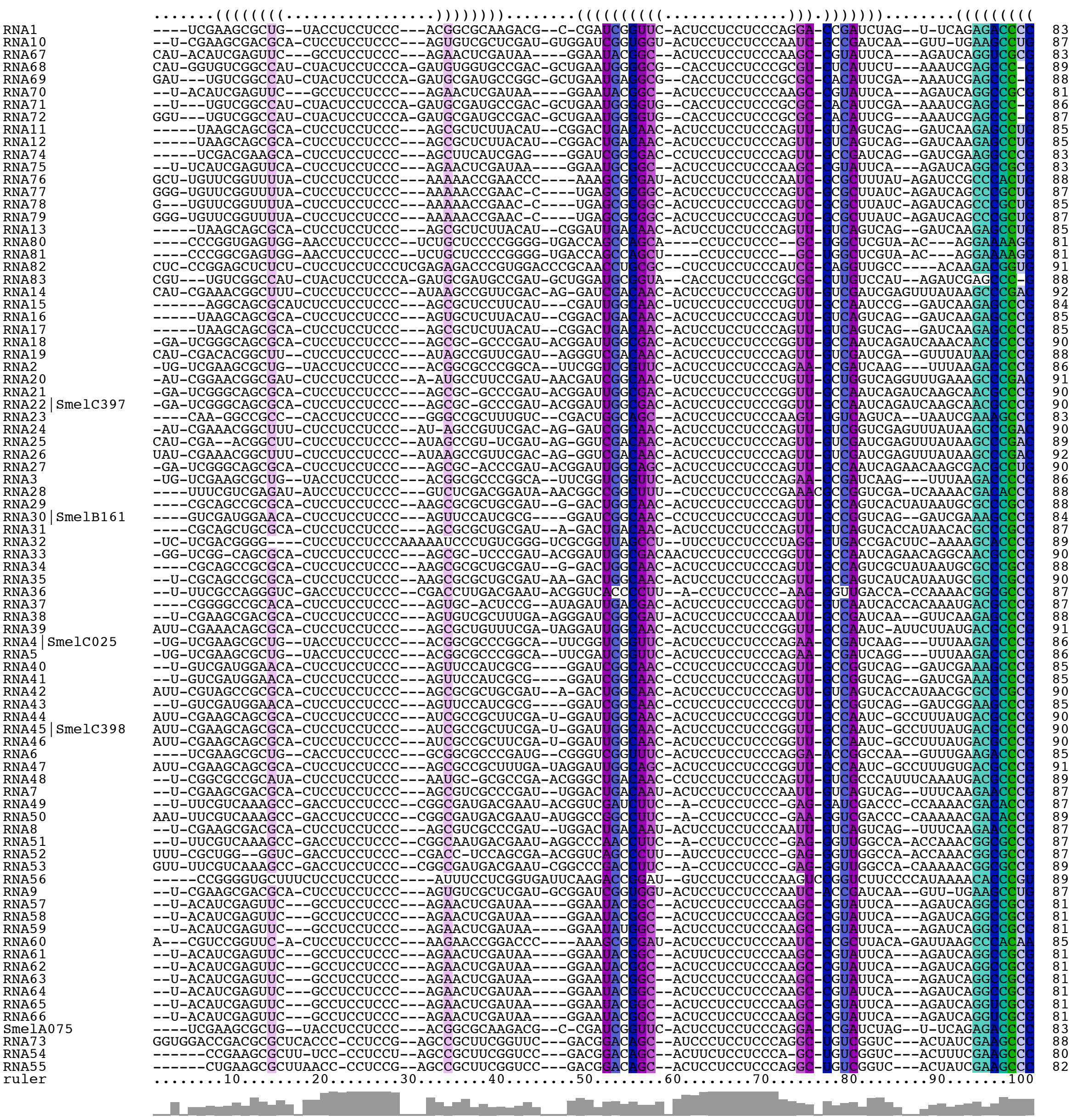

RNA Family Model of SmelA075. *Cont.*

(a)

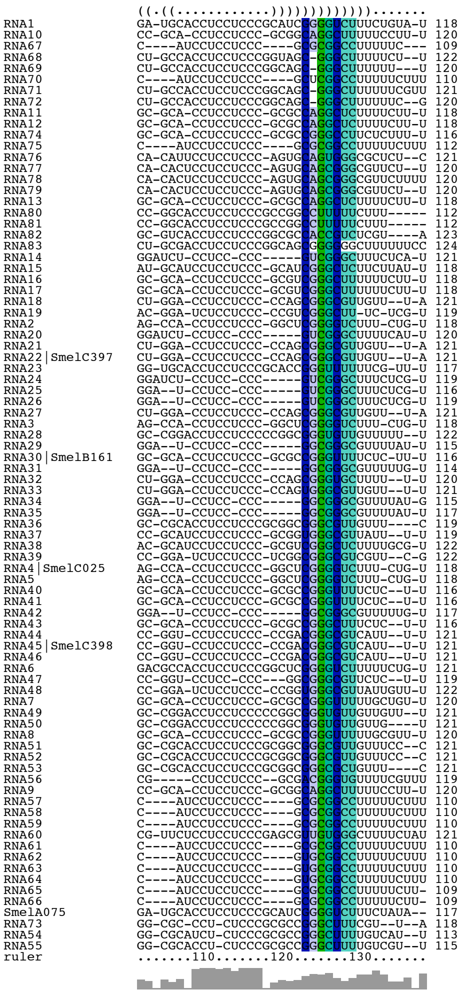

(b)

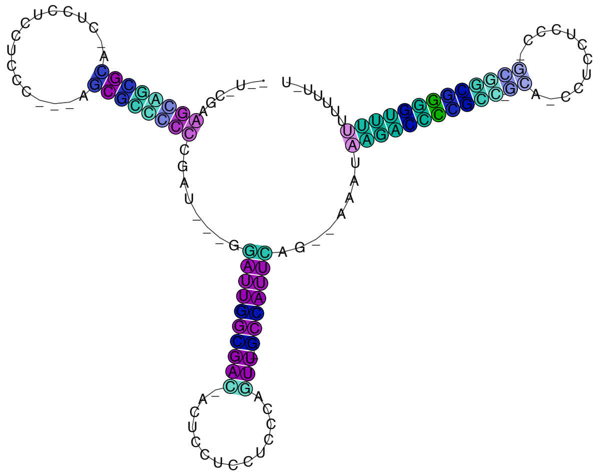

RNA Family Model of SmelA099. (**a**) Alignment of $RFM_{SmelA099}$ sequences (SCI = 0.87); (**b**) Consensus secondary structure (minimum free energy -60.52 kcal/mol) for the alignment shown in (**a**).

(a)

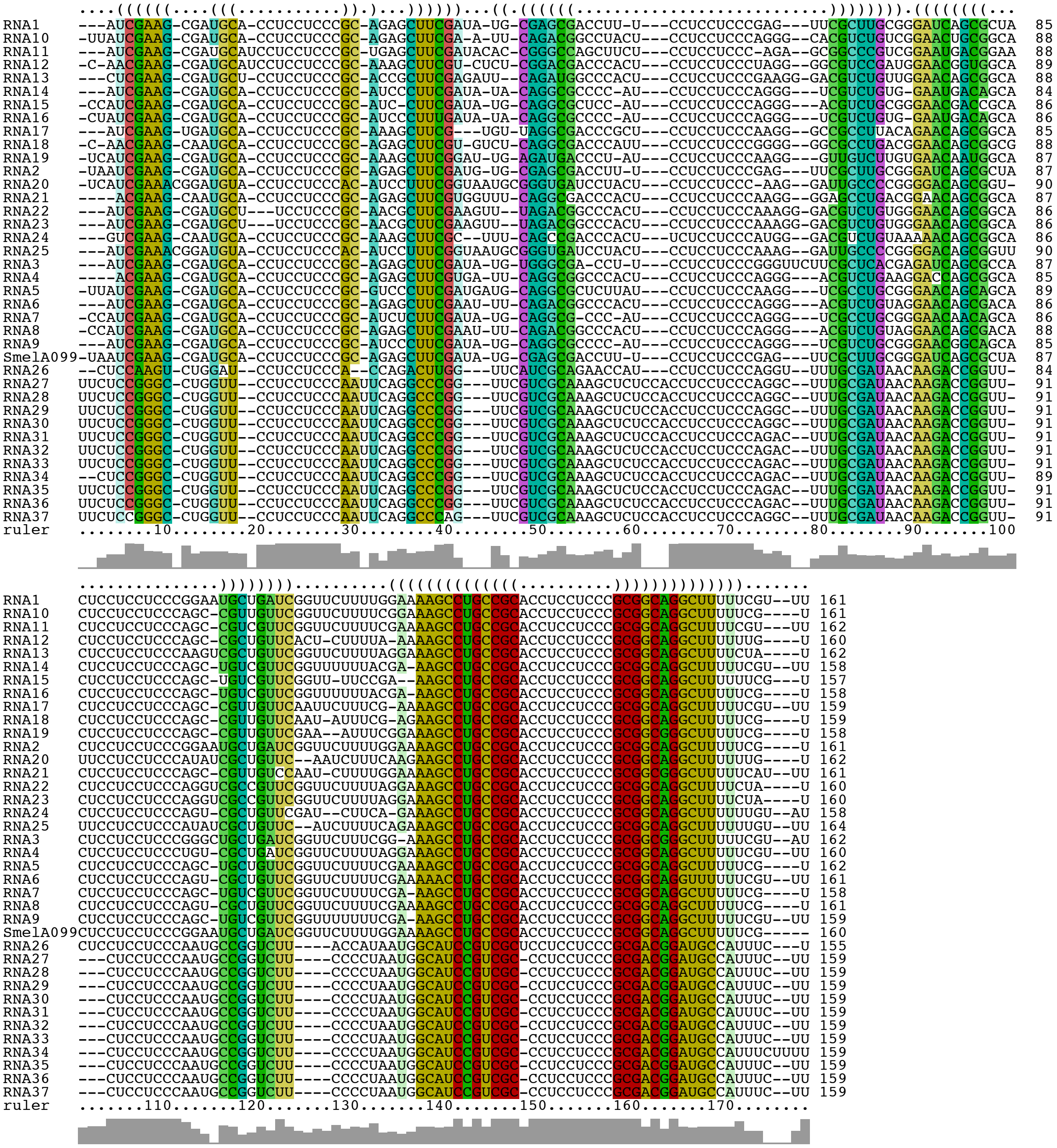

RNA Family Model of SmelA099. *Cont.*

(b)

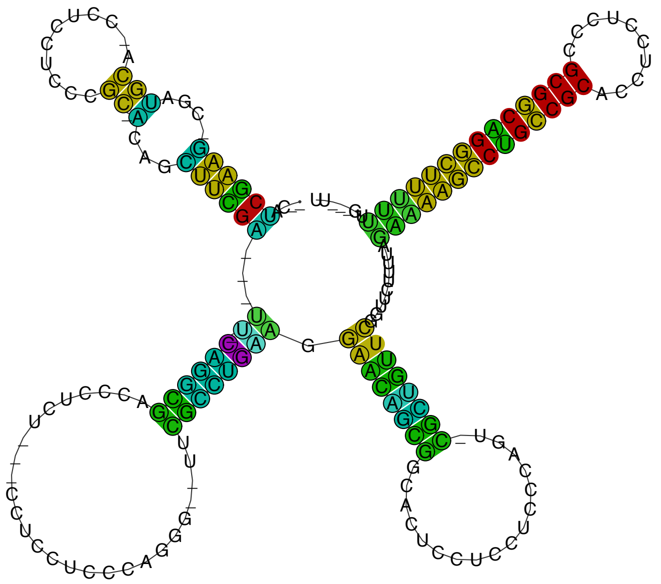

RNA Family Model of SmelB003. (**a**) Alignment of $RFM_{SmelB003}$ sequences (SCI = 0.99); (**b**) Consensus secondary structure (minimum free energy -56.04 kcal/mol) for the alignment shown in (**a**).

(a)

```
          .((((((((..(((....(((..((.......)).))))))))))))))((((((((((((((((.((................)).)))))))))))))
RNA1      UGCGCGUUCACGGGAUAACGGAAAG-CCCCAACUGCCGCCCGGGCGCGCGAGGGUCGCAGAGCGACGCCGCCCCAGCCCCAUCUGCAUCGCUCUGCGACC  99
RNA2      UGCGCGUUCACGGGAUAACGGAAAG-CCCCAACUGCCGCCCGGGCGCGCGAGGGUCGCAGAGCGACGCCGCCCCAGCCCCAUCUGCAUCGCUCUGCGACC  99
SmelB003  UGCGCGUUCACGGGAUAACGGAAAG-CCCCAACUGCCGCCCGGGCGCGCGAGGGUCGCAGAGCGACGCCGCCCCAGCCCCAUCUGCAUCGCUCUGCGACC  99
RNA3      UGCGCGCCG---GGACAAUGGACAAGCCCCAAUUGCCAUCCCGGCGCGCGAGGGUCGCAGAGCGGCGC-GCCCCAGCCCCAUCUGUAUCGCUCUGCGACC  96
ruler     .........10........20........30........40........50........60........70........80........90.......100

          ))).......
RNA1      CUCGCCUUG 108
RNA2      CUCGCCUUG 108
SmelB003  CUCGCCUUG 108
RNA3      CUCGCCUGG 105
ruler     .......110
```

(b)

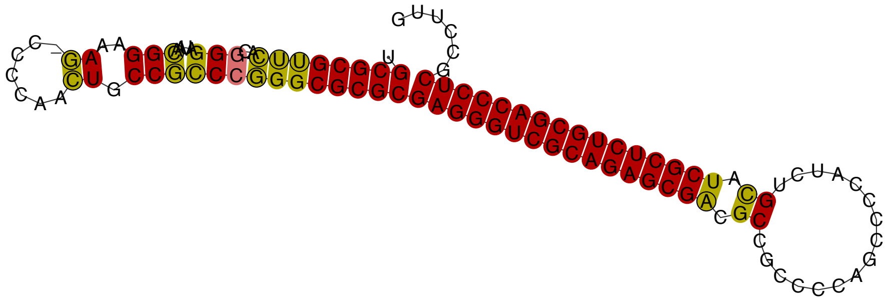

RNA Family Model of SmelB008. (**a**) Alignment of $RFM_{SmelB008}$ sequences (SCI = 1.00); (**b**) Consensus secondary structure (minimum free energy -34.40 kcal/mol) for the alignment shown in (**a**).

(a)

```
          ((((((..........))))))...((((.......))))..(((((((((((((........))))))))))).))
RNA1      GUCGCUUCUGUCCCCCAGCGACAUUCAGGACCCCAACCUGUUGCAGUGCCCGCGUCAUCCCCUGCGCGGGCACUCGC 77
RNA2      GUCGCUUCUGUCCCCCAGCGACAUUCAGGACCCCAACCUGUUGCAGUGCCCGCGUCAUCCCCUGCGCGGGCACUCGC 77
RNA3      GUCGCUUCUGUCCCCCAGCGACAUUCAGGACCCCAACCUGUUGCAGUGCCCGCGUCAUCCCCUGCGCGGGCACUCGC 77
SmelB008  GUCGCUUCUGUCCCCCAGCGACAUUCAGGACCCCAACCUGUUGCAGUGCCCGCGUCAUCCCCUGCGCGGGCACUCGC 77
ruler     .........10........20........30........40........50........60........70.......
```

(b)

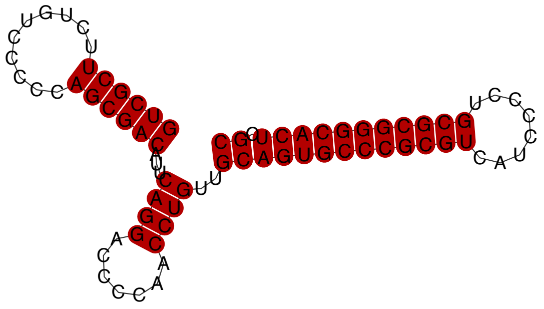

RNA Family Model of SmelB009. (**a**) Alignment of $RFM_{SmelB009}$ sequences (SCI = 1.02); (**b**) Consensus secondary structure (minimum free energy -34.61 kcal/mol) for the alignment shown in (**a**).

(a)

```
          ............(((((.((((((....))))).).).))))(((((((((((((((.....))))))))))))))).....
RNA1      UUCCCACCUAUACAAGGACCUAAACCUUUUUAGCGACACUUGAGGCGGGGCCUCGCCGCUU-GGCGGGGUCCCGCUU-GCGU 80
RNA2      UUCCCACCUAUACAAGGACCUAAACCUUUUUAGCGACACUUGAGGCGGGGCCUCGCCGCUUCGGCGGGGUCCCGCUU-GCGU 81
SmelB009  UUCCCACCUAUACAAGGACCUAAACCUUUUUAGCGACACUUGAGGCGGGGCCUCGCCGCUU-GGCGGGGUCCCGCUU-GCGU 80
RNA3      UUCCCACCUAUACAAGAACCUAGACCUUUUCAGCGAUAUCUGGGGCGGGGUUUUGCCACGAUGGCAGAACCCCUCCUUGGAU 82
ruler     .........10........20........30........40........50........60........70........80..
```

(b)

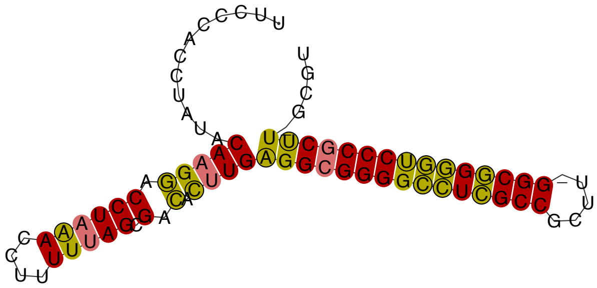

RNA Family Model of SmelB033. (**a**) Alignment of $RFM_{SmelB033}$ sequences (SCI = 0.67); (**b**) Consensus secondary structure (minimum free energy -24.07 kcal/mol) for the alignment shown in (**a**).

(a)

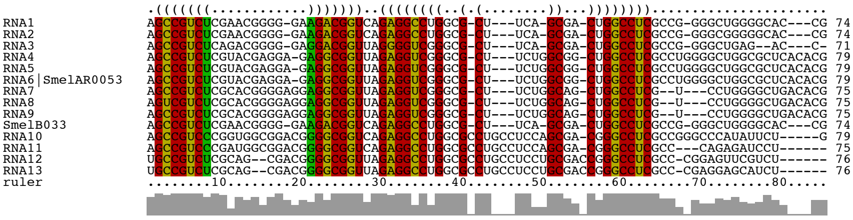

(b)

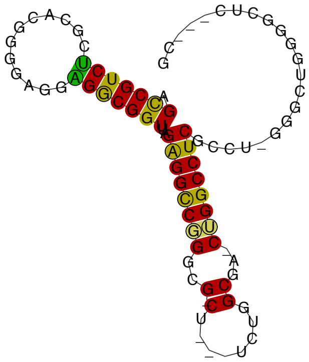

RNA Family Model of SmelB044. (**a**) Alignment of $RFM_{SmelB044}$ sequences (SCI = 0.76); (**b**) Consensus secondary structure (minimum free energy -41.37 kcal/mol) for the alignment shown in (**a**).

(a)

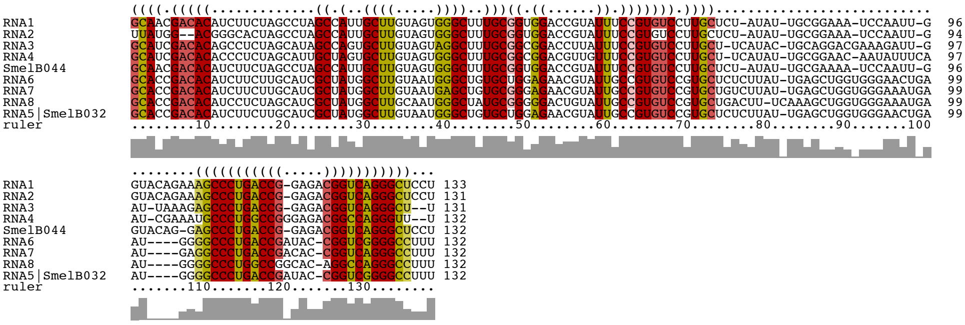

(b)

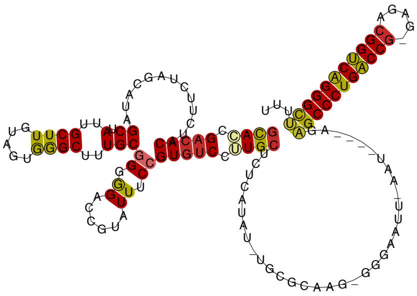

RNA Family Model of SmelB053. (**a**) Alignment of $RFM_{SmelB053}$ sequences (SCI = 0.32); (**b**) Consensus secondary structure (minimum free energy -16.88 kcal/mol) for the alignment shown in (**a**).

(a)

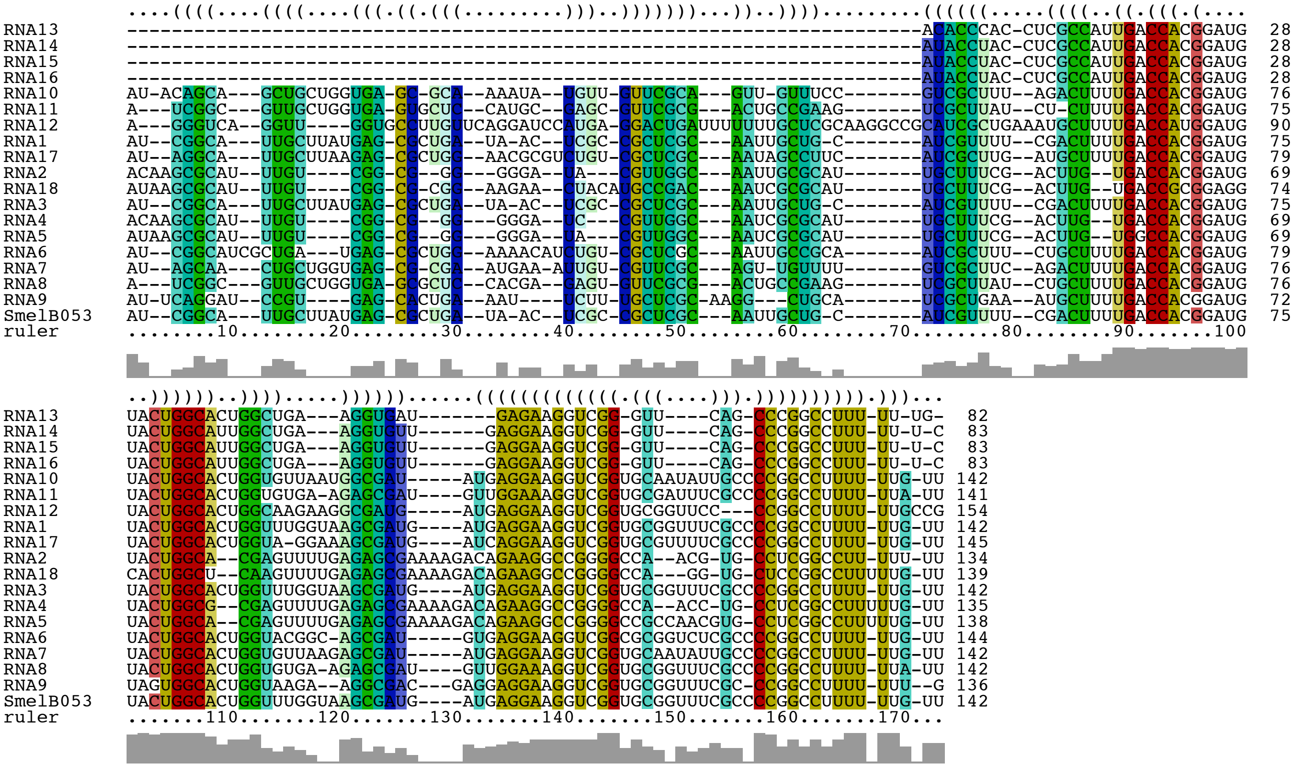

(b)

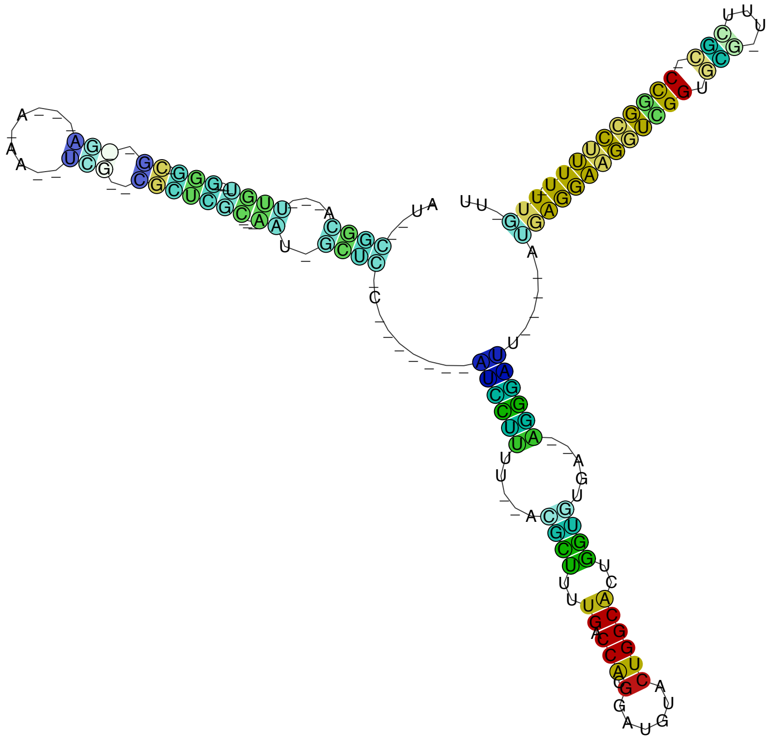

RNA Family Model of SmelB064. (**a**) Alignment of $RFM_{SmelB064}$ sequences (SCI = 0.91); (**b**) Consensus secondary structure (minimum free energy -32.97 kcal/mol) for the alignment shown in (**a**).

(a)

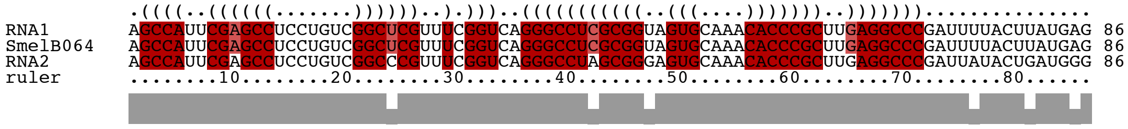

(b)

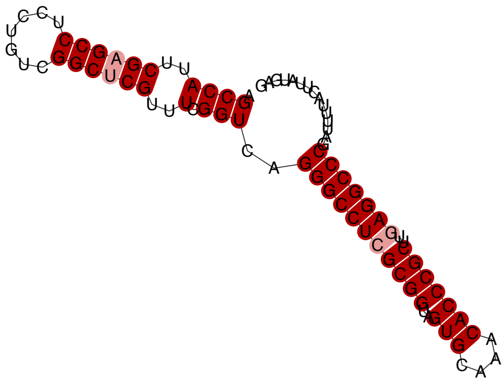

RNA Family Model of SmelB075. (**a**) Alignment of $RFM_{SmelB075}$ sequences (SCI = 1.00); (**b**) Consensus secondary structure (minimum free energy -15.81 kcal/mol) for the alignment shown in (**a**).

(a)

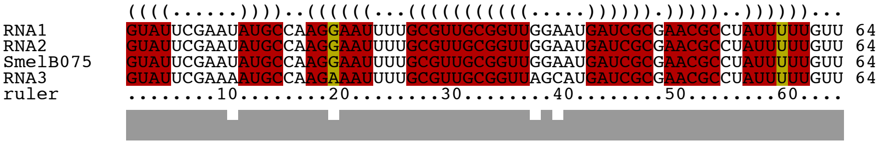

(b)

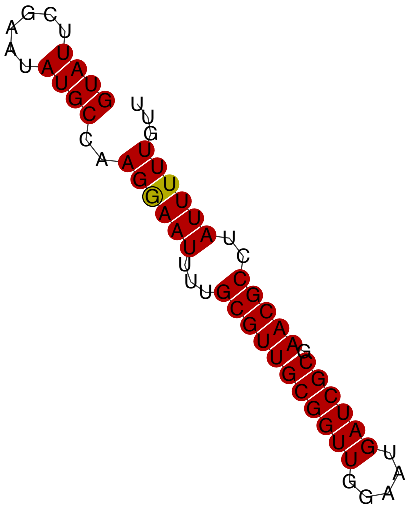

RNA Family Model of SmelB095. (**a**) Alignment of $RFM_{SmelB095}$ sequences (SCI = 0.89); (**b**) Consensus secondary structure (minimum free energy -43.39 kcal/mol) for the alignment shown in (**a**).

(a)

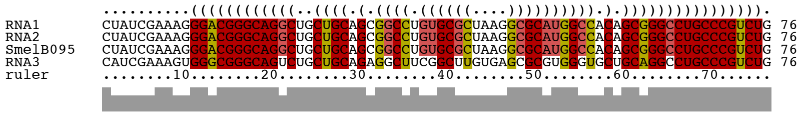

(b)

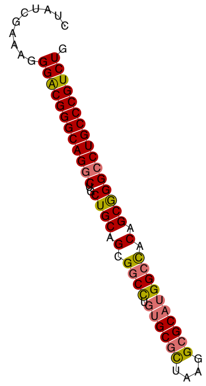

RNA Family Model of SmelB126. (**a**) Alignment of $RFM_{SmelB126}$ sequences (SCI = 0.7); (**b**) Consensus secondary structure (minimum free energy -27.44 kcal/mol) for the alignment shown in (**a**).

(a)

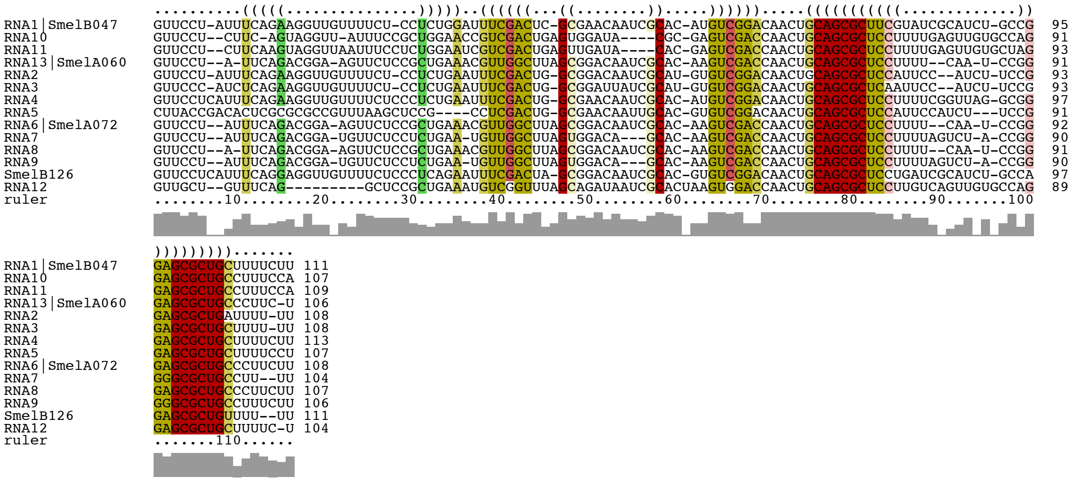

(b)

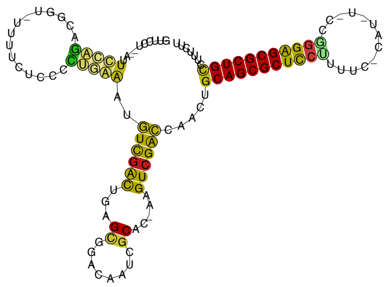

RNA Family Model of SmelC023. (**a**) Alignment of $RFM_{SmelC023}$ sequences (SCI = 1.11); (**b**) Consensus secondary structure (minimum free energy -78.06 kcal/mol) for the alignment shown in (**a**).

(a)

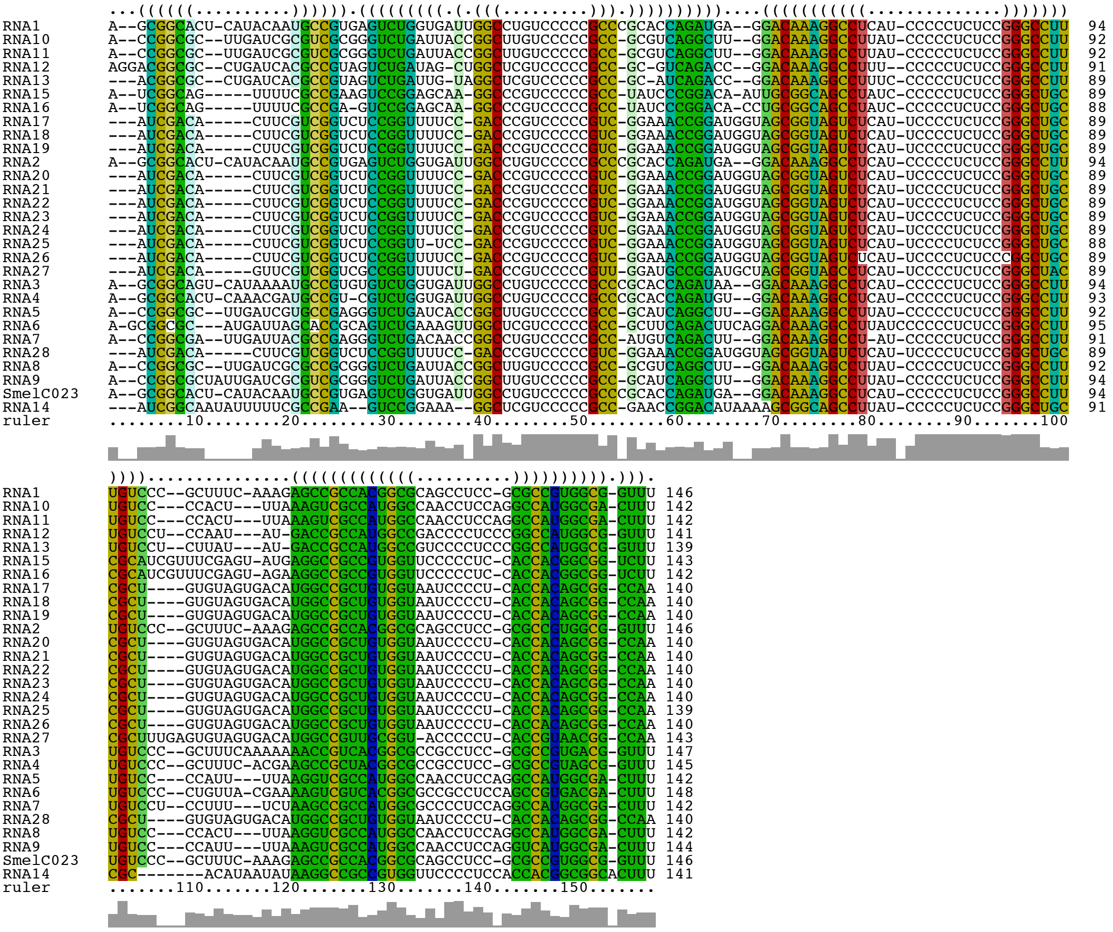

(b)

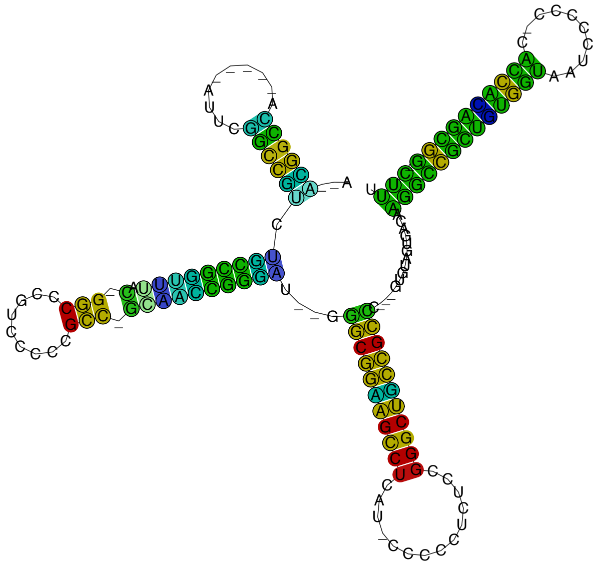

RNA Family Model of SmelC032. (**a**) Alignment of $RFM_{SmelC032}$ sequences (SCI = 1.00); (**b**) Consensus secondary structure (minimum free energy -38.80 kcal/mol) for the alignment shown in (**a**).

(a)

```
            ((.(.(((((((......))))))))...(.((.....)).)))......((((.((((((....)))))).))))...
RNA1        GCAGUCCGGCCGCUGAAAUGGCCGGCGGAGGCGGCUCACGGCGCAAAAAAGGGGAGCGGACAUAGGUCCGCACCCCAUU 79
SmelC032    GCAGUCCGGCCGCUGAAAUGGCCGGCGGAGGCGGCUCACGGCGCAAAAAAGGGGAGCGGACAUAGGUCCGCACCCCAUU 79
RNA2        GCAGUCCGGCCGCUGAAAUGGCCGGCGGAGGCGGCUCACGGCGCAAAAAAGGGGAGCGGACAUAGGUCCGCACCCCAUU 79
ruler       .........10........20........30........40........50........60........70.........
```

(b)

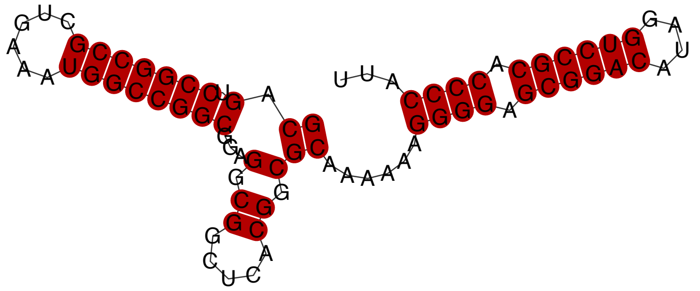

RNA Family Model of SmelC055. (**a**) Alignment of $RFM_{SmelC055}$ sequences (SCI = 1.04); (**b**) Consensus secondary structure (minimum free energy -38.96 kcal/mol) for the alignment shown in (**a**).

(a)

```
            .......(((((....)))))((((((((((((((((....))))))))))))))))......
RNA1        GAGUU--UUGGCGGGGGCCAGGAGCCGGGUGCGUUUCGAAAGAGACGCAUCCGGCUU-UAUGU 60
RNA2        GAGUU--UUGGCGGGGGCCAGGAGCCGGGUGCGUUUCGAAAGAGACGCAUCCGGCUU-UAUGU 60
RNA3        CAGUU--UUGGCGGGGGCCAGGAGCCGGGUGCGUUUCGAAGGAGACGCAUCCGGCUU-UAUGU 60
SmelC055    GAGUU--UUGGCGGGGGCCAGGAGCCGGGUGCGUUUCGAAAGAGACGCAUCCGGCUU-UAUGU 60
RNA4        GAGUUCAUUGGCGGGGGCCAGGGGCCGCAUGCGUUUCGCAAGAGACGCAUGCGGCUUUUUUGU 63
ruler       .........10........20........30........40........50........60...
```

(b)

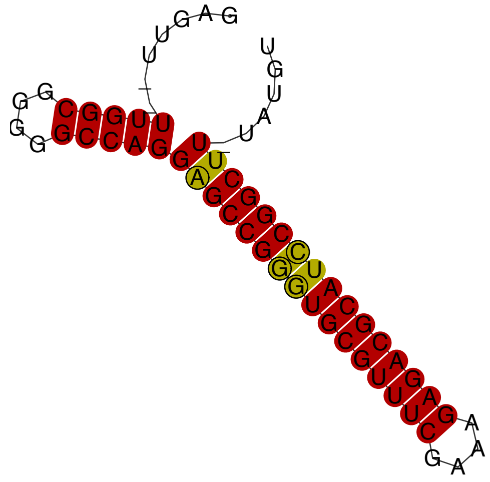

RNA Family Model of SmelC151. (**a**) Alignment of $RFM_{SmelC151}$ sequences (SCI = 1.02); (**b**) Consensus secondary structure (minimum free energy -36.37 kcal/mol) for the alignment shown in (**a**).

(a)

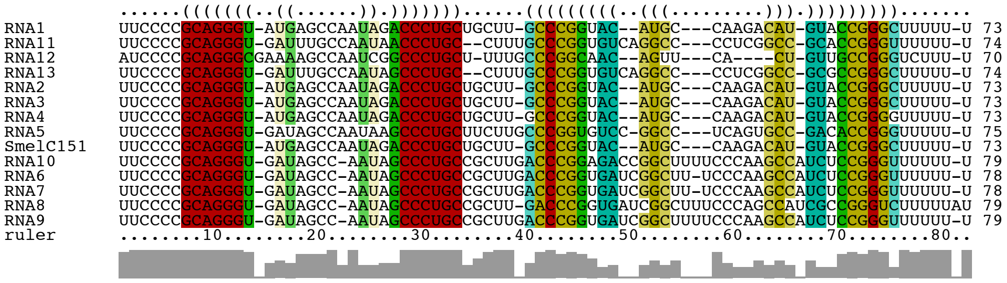

(b)

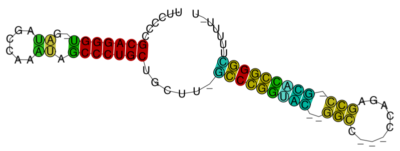

RNA Family Model of SmelC165. (**a**) Alignment of $RFM_{SmelC165}$ sequences (SCI = 0.71); (**b**) Consensus secondary structure (minimum free energy -43.24 kcal/mol) for the alignment shown in (**a**).

(a)

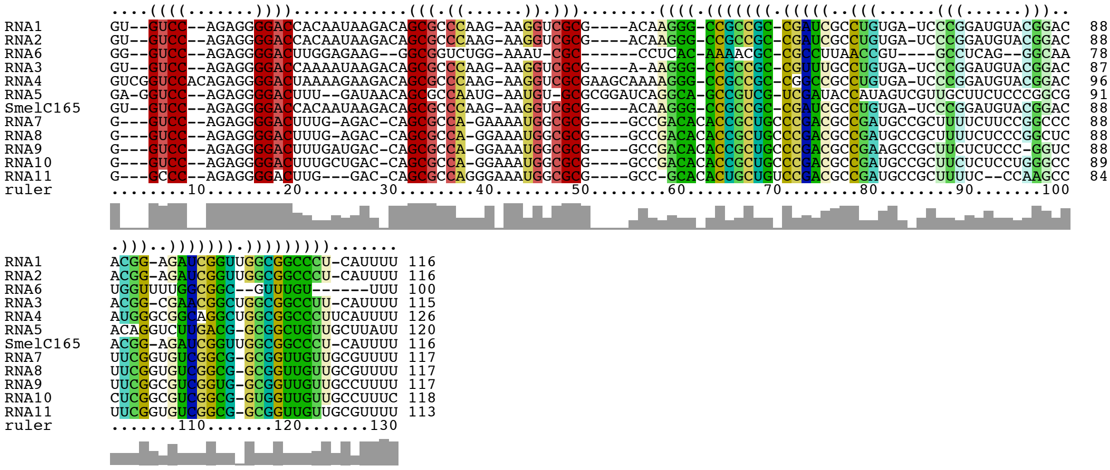

(b)

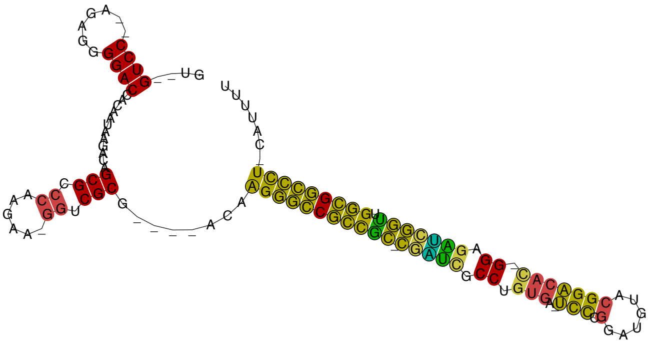

RNA Family Model of SmelC289. (**a**) Alignment of $RFM_{SmelC289}$ sequences (SCI = 0.9); (**b**) Consensus secondary structure (minimum free energy -48.93 kcal/mol) for the alignment shown in (**a**).

(a)

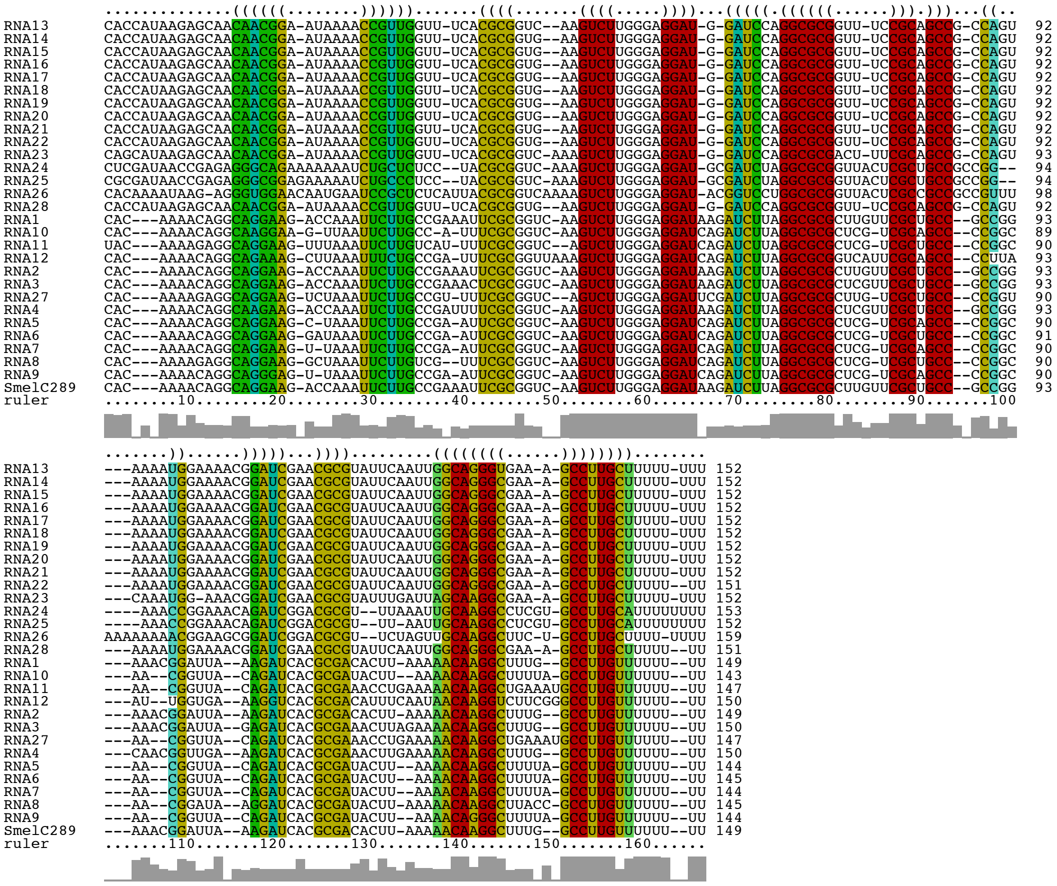

(b)

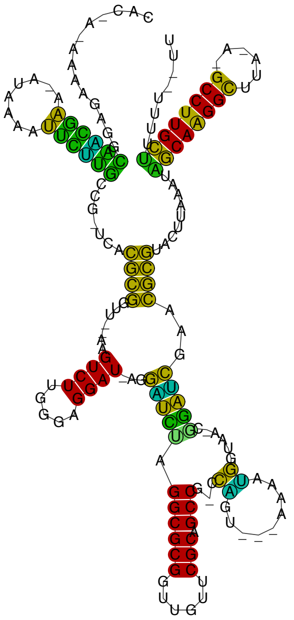

RNA Family Model of SmelC291. (**a**) Alignment of $RFM_{SmelC291}$ sequences (SCI = 0.55); (**b**) Consensus secondary structure (minimum free energy -42.63 kcal/mol) for the alignment shown in (**a**).

(a)

```
             ......................................((((((((..((..(((((............))))))).)))))))).((((((((((((.(
RNA17        -CCAUCGAAGCGCAUUUGGC-GACUUGACUGG-AAGUUACCCUACGGAGUGCUCAGGUCCGCCGCAUCUCCUGGGUCCGUAGGGUCGGCCGGCGCGCAAG  97
RNA18        GUCGGUUGGGGUAGCUUUCCCGACCUAUCCG----CUUGCCCUGCGGAAUGCUCGGGUCCGCCGCAUCUCCUGGGCCCGCAGGUCUGGUCGGCG-GGCGA  95
RNA19        GCGCAUAUCGAUGCUCGUCC-GACAUGACUG--------CCCUGCGGAGUG-CCAGGUCCGCCGCAUCUCCUGGGUCCGUAGGGUUGGCCGGCGCGCAAA  90
RNA20        --CCGAGGCACCGCCAGUCCCGCCAUCAGGA-----CAACCCUGCGGAGUGCUCAGGUCCGCCGCAUCUCCUGGGUCCGUGGGGUUUGGCCGGUGGGUGA  93
RNA21        --GCG--CCUUGGCGCGCAACGCC-U-GUUC-----UAGCCCUGCGGAGUGCCUAGGUCCGCCGCAUCUCCUGGGUCCGUGGGGUUCGGCCGGCGGGUGG  89
RNA22        -UCGAGU-GUUUCCUCUUACUUA--UAAGCA----GUUACCCUGCGGAGUGCUCGGGUCCGCCGCAUCUCCUGGGUCCGUAGGGCA--UACGAC-CGAAG  89
RNA10        CAUAAAAGA-AGCGCUCCCGCAGAGGAGCUG-U-CGAUACCCCAUG--AUGCUCAGGUCCGCCGCAUCUCCUGGGU-CGUGGGGUCGGUCGGCUAGUAGG  94
RNA11        G-UCAG-----GGGCUGGUAUUGCGUAGUU--U-GAAUGCCCCAUG--AUGCUCAGGUCCGCCGCAUCUCCUGGAU-CGUGGGGUCGGUCGGCUGACGAU  88
RNA12        G-UCAG-----GGGCUGGUAUUGCGUAGUU--U-GAAUGCCCCAUG--AUGCUCAGGUCCGCCGCAUCUCCUGAAU-CGUGGGGUCGGUCGGCUGACGAU  88
RNA13        G-CAGUA-AAAACUGACGAAUAACAGGUUUA-ACCAAUACCCCAUG--GUGCUCAGGUCCGCCGCAUCUCCUGGGU-CGUGGGGUUGGUCGGCAGGUGUG  94
RNA14        G-GUGGCUGGACAUAAUG--UACGGUUGCCGCUCAAAUACCCCAUG--AUGCUCAGGUCCGCCGCAUCUCCUGGGUCCGUGGGGUUGGCCGGUUGGUG-C  94
RNA15        ACGAGGAUAGACCGCCGGC-UCCGGUCCCUGGG--AAUACCCCAUG--AUGCUCAGGUCCGCCGCAUCUCCUGGGUCCGUGGGGUCGGCCGGCCGGCG-C  94
RNA16        GCGAGGGUAGAUAGCCGGC-U-CGAUCCCUAGA--AAUACCCCAUG--AUGCUCAGGUCCGCCGCAUCUCCUGGGUCCGUGGGGUCGGCCGGCCGGCG-C  93
RNA1         GCGAGGAAG-GAAACU----UCCAC-AGCAU-UCGGUUACCCCAUG--AUGCUCAGGUCCGCCGCAUCUCCUGGGU-CGUGGGGUCGGUCGGCUGGCCUC  90
RNA2         GCGAGGAAG-GAAACU----UCCAC-AGCAU-UCGGUUACCCCAUG--AUGCUCAGGUCCGCCGCAUCUCCUGGGU-CGUGGGGUCGGUCGGCUGGCCUC  90
RNA3         GCGAGGAAG-GAAACU----UCCAC-AGCAA-UCGGUUACCCCAUG--AUGCUCAGGUCCGCCGCAUCUCCUGGGU-CGUGGGGUCGGUCGGCUGGCCUC  90
RNA4         UUAAGGAAGUGUGACU----UCCACGAUCGA-UCGGUUACCCCAUG--AUGCUCAGGUCCGCCGCAUCUCCUGGGU-CGUGGGGUCGGUCGGCUGGAUUC  92
RNA5         UCCCGUACAGAGAUAGCCCGCAA-GGGUUUG-A-GAAUACCCCAUG--AUGCUCAGGUCCGCCGCAUCUCCUGGGU-CGUGGGGUCGGUCGGCUGGCAGG  94
RNA6         CAUAAGAGA-AGCGCUCCCGUUGAGGAGUUG-U-CGAUACCCCAUG--AUGCUCAGGUCCGCCGCAUCUCCUGGGU-CGUGGGGUCGGUCGGCUAGUAGG  94
RNA7         ---AAGAGA-AGCGCUCCCGUUGAGGAGCUG-U-CGAUACCCCAUG--AUGCUCAGGUCCGCCGCAUCUCCUGGGU-CGUGGGGUCGGUCGGCUAGUAGG  91
RNA8         CAUAAGAGA-AGCGCUCCCGCUGAGGAGUUG-U-CGAUACCCCAUG--AUGCUCAGGUCCGCCGCAUCUCCUGGGU-CGUGGGGUCGGUCGGCUAGUAGG  94
RNA9         ----ACAGA-AGCGCUCCCGUAGAGGAGCUG-UCCGAUACCCCAUG--AUGCUCAGGUCCGCCGCAUCUCCUGGGU-CGUGGGGUCGGUCGGCUGGUAGG  91
SmelC291     GCGAGGAAG-GAAACU----UCCAC-AGCAU-UCGGUUACCCCAUG--AUGCUCAGGUCCGCCGCAUCUCCUGGGU-CGUGGGGUCGGUCGGCUGGCCUC  90
ruler        .........10........20........30........40........50........60........70........80........90.......100

             ((..............................))).))))))))))..))..................................................
RNA17        U-AGUU-GGAGUGGCCUCCUCGAAACGCU---ACU-UGC-CGCCGG--CCACCU-UCA-GGCCUCGCCCAUGUGCGAGU-----U--GGU------UGCG 173
RNA18        UGGCUCGGAAGGCCUCUCCGUC------C---CAUCGCC-CGCCGAUUCCAGAG-UAGCU----CUCUGUU-------UCC--CU--GAU--AACCGUCA 167
RNA19        UGGCUUAGGAGUGGCCUCCUCGAAACGGC---CAUCUGC-CGCCGG--CCACCU-UCG-UG---AGGAAAUCGUC---------U--CGU------GUCA 162
RNA20        GC--UUGCGGGAGGCCUCCCCGC----UU---UCUCCCC-ACCGGC--CAGCCUAU--CUUC--CGCAGACGAUC--------AC--ACCCCAUUGGAAG 167
RNA21        ACGAUCACGGGUGGCCUCCCCGU----CC---GUCCACC-GCCGGC--CA-CCUCUCCCUGA--CAUGUCUGAUC--------CU--GCUGC-CGCCCCG 165
RNA22        CCUCUUGU-CGUCGCAAACAUGA-----U---GGUUUCG-GG-CAAUAGCACGU-UGGAUCACGCCCUCUUGAUCCGAUGGCGCUCAGAUCAGCUCCGCA 177
RNA10        CAAGUCAU--AACGGUUCCUCGUGCCGUU-U-UGCAUGCUGGCCGA--CCCCC--U-U-UCAAGAAAACGCCGCCACUUC---CGGAAGA------UGGA 175
RNA11        GUCCGAAU----CGGUUCCUCGUGCCGUGAUCACAUCGUUAGCCGA--CCGCCUUA-UCUCCGAAAAUAUUCUGUGCUUG----UGCUGC------CGCU 171
RNA12        GUCCGAAU----CGGUUCCUCGUGCCGUAAUCACAUCGUUGGCCGA--CCGCCUUC-U-UCCCGAAAAAGCCUGU--UGG----UGGCGU------UCGU 168
RNA13        CGA-UUAUCAAGCGGUUCCUCGUGCCGUUAU-UGCAUGCCCGCCGA--CCCCC--A-U-UCCAACGCCGCU-UCCCAACGC--CGCUUCC------UUGU 177
RNA14        UGGUG-UU-AGUGGCCUCCUCGUA-CCGCAGCCAGUUGCCAGCCGG--CCCCC--A-ACAGCGCGCCA----AUCUGCCCUC--UCAUCU------U--- 171
RNA15        UGGCACAU-AGUGGCCUCCUCGUA-CCGCUGCCAAACGCCGACCGG--CCCCC--U-UCUUUCCAA-UGAACAUCAGAGCU---CCGUGC------CAAA 177
RNA16        UGGCACAU-AGUGGCCUCCUCGUA-CCGCUGCCAAACGCCGACCGG--CCCCC--U-UCUUUCAAACAGAACAUCAGAACU---UCGUGC------CAAG 177
RNA1         GGACU--U--GCGGAUUCCUCGUG-CUGCA-ACCGG-ACCAGUCGA--CCCCC--A-U-UCG-AAACGCCGCUUCGAAAGAGGCGGCG-U------U--U 167
RNA2         GGACU--U--GCGGAUUCCUCGUG-CUGCA-ACCGG-ACCAGUCGA--CCCCC--A-U-UCG-AAACGCCGCUUCGAAAGAGGCGGCG-U------U--U 167
RNA3         GGAUU--U--GCGGAUUCCUCGUG-CUGCA-ACCGGAACCAGCCGA--CCCCC--A-U-UCGAAAACGCCGCUUCGAAAGAGGCGGCG-U------U--U 169
RNA4         CGACG--UCCGCGGAUUCCUCGUG-CCGCAGUCGGAGCCCAGCCGA--CCCCC--U-U-UCG-GAACGCCGCUCCGAAGGAGGCGGCGUU------U--U 174
RNA5         CAA-UU-UCAAGCGGUUCCUCGUGCCGUU-U-UGCAGGCUAGCCGG--CCCCCGAU-U-UAAAACGCCGCUCUUCCUAUGGAAGGCGGCG------UU-U 179
RNA6         CAAGUUAU--AGCGGUUCCUCGUGCCGUU-U-UGCAUGCUGGCCGA--CCCCC--U-U-UCAAGAAAACGCCGCCACUUC---CCGAAGA------UGGA 175
RNA7         CAAGUUAU--AGCGGUUCCUCGUGCCGUU-U-UGCAUGCUGGCCGA--CCCCC--U-U-UCAAGAAAACGCCGCCACUUC---CCGAAGA------UGGA 172
RNA8         CAAGUUAU--AGCGGUUCCUCGUGCCGUU-U-UGCAUGCUGGCCGA--CCCCC--U-U-UCAAGAAAACGCCGCCACUUC---CCGAAGA------UGGA 175
RNA9         CAAGUCAU--AACGGUUCCUCGUGCCGUU-U-UGCAUGCUGGCCGA--CCCCC--U-U-UCAGGAAAACGCCGUCACUUC---CCGAAGA------UGGA 172
SmelC291     GGACU--U--GCGGAUUCCUCGUG-CUGCA-ACCGG-ACCAGUCGA--CCCCC--A-U-UCG-AAACGCCGCUUCGAAAGAGGCGGCG-U------U--U 167
ruler        .......110.......120.......130.......140.......150.......160.......170.......180.......190.......200

             ....
RNA17        C--U 175
RNA18        UCAU 171
RNA19        ---U 163
RNA20        CGCG 171
RNA21        UGC- 168
RNA22        UCAU 181
RNA10        AGGG 179
RNA11        U--A 173
RNA12        A--U 170
RNA13        CCGG 181
RNA14        ---- 171
RNA15        GCA- 180
RNA16        GC-- 179
RNA1         UCGU 171
RNA2         UCGU 171
RNA3         UCGA 173
RNA4         UCGU 178
RNA5         ---- 179
RNA6         AGGG 179
RNA7         AGGG 176
RNA8         AGGG 179
RNA9         AGGG 176
SmelC291     UCGU 171
ruler        .......210
```

RNA Family Model of SmelC291. *Cont.*

(b)

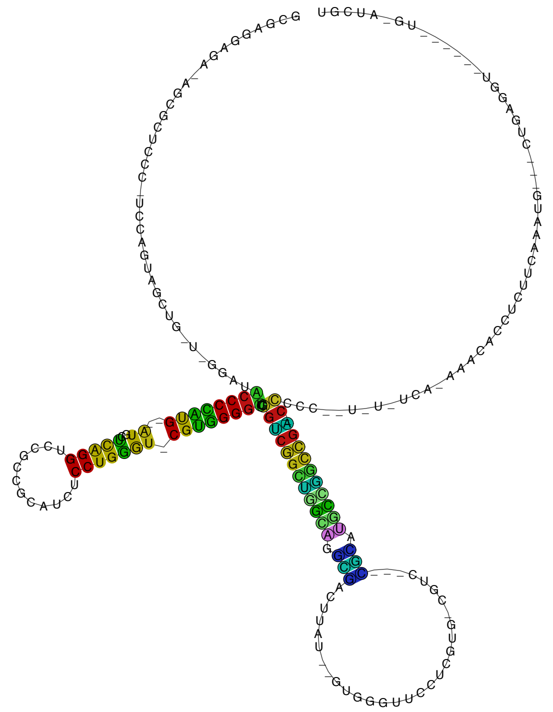

RNA Family Model of SmelC416. (**a**) Alignment of $RFM_{SmelC416}$ sequences (SCI = 0.98); (**b**) Consensus secondary structure (minimum free energy -43.52 kcal/mol) for the alignment shown in (**a**).

(a)

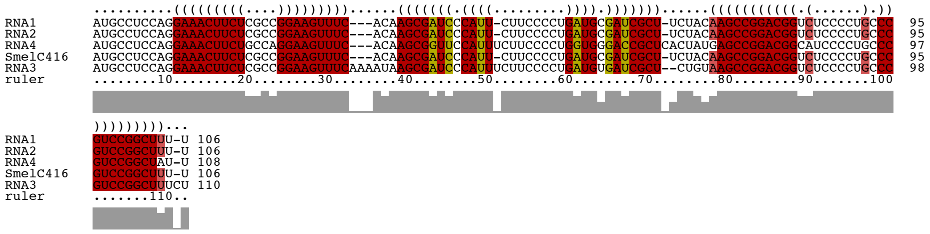

(b)

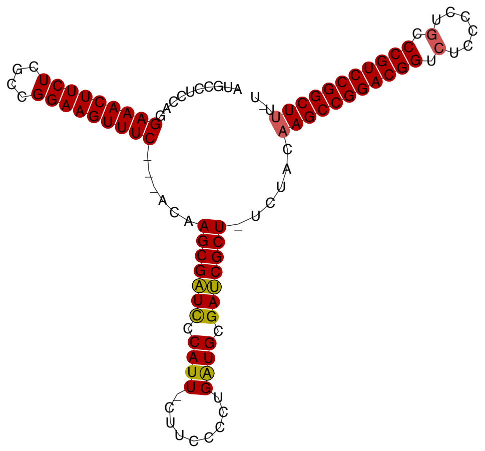

RNA Family Model of SmelC434. (**a**) Alignment of $RFM_{SmelC434}$ sequences (SCI = 0.99); (**b**) Consensus secondary structure (minimum free energy -59.35 kcal/mol) for the alignment shown in (**a**).

(a)

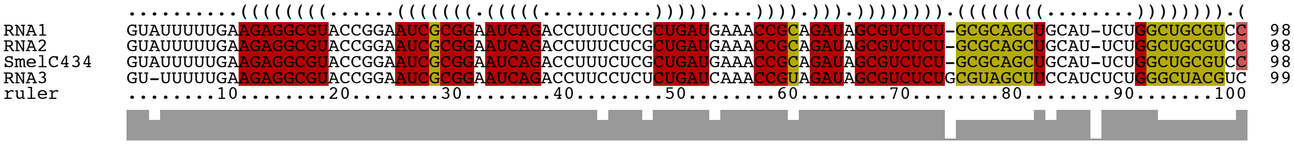

(b)

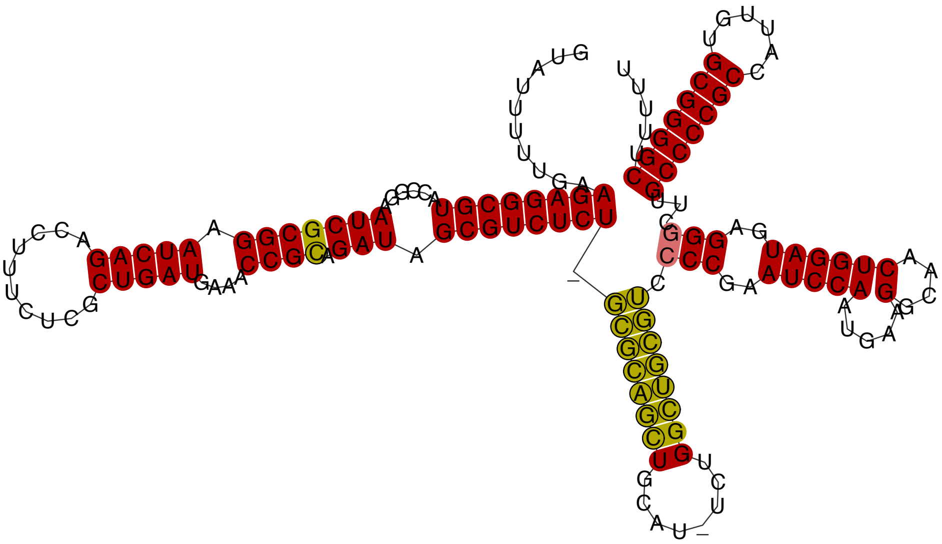

RNA Family Model of SmelC500. (**a**) Alignment of $RFM_{SmelC500}$ sequences (SCI = 1.01); (**b**) Consensus secondary structure (minimum free energy -101.60 kcal/mol) for the alignment shown in (**a**).

(a)

```
         ..((((((...((((((((((....))))).)))))))))))..(((((((((((((..(((...((((((((((((((((((...))))))))))...)
RNA1     UUCACGGCGAUGCUGCGCGUUCC-AAACGCCGCGGCGCCGUGUACGGCCGGCGAUGC--GCCUUCCGCGUCUCCCGAAUCCGCGAGGCGGAUUCGGCGUG  97
RNA2     UUCACGGCGAUGCUGCGCGUUCC-AAACGCCGCGGCGCCGUGUACGGCCGGCGAUGC--GCCUUCCGCGUCUCCCGAAUCCGCGAGGCGGAUUCGGCGUG  97
RNA3     CUCACGGCGAUCCAGCGCGUCCCAAGACGCUGCGGCGCCGUGUGCGGCCGGCGAUGC--GCCUCCGCGUCUCCCCGAACCCGCCAGGCGGUUUCGGAUUG  98
SmelC500 UUCACGGCGAUGCUGCGCGUUCC-AAACGCCGCGGCGCCGUGUACGGCCGGCGAUGC--GCCUUCCGCGUCUCCCGAAUCCGCGAGGCGGAUUCGGCGUG  97
RNA4     UUACGGCG-ACCUGCGCGUCUUC-AAGACGCCGCGGCGUCGUAUGCGGCCGGCGAUGCGCCUUUCGCGUCGACCCGAAUCCGCAGGGCGGUUCGGAAGCG  98
ruler    .........10........20........30........40........50........60........70........80........90.......100

         )..))))))..........((((((..(.......)(((.......))).......)))))))))...))))))))))))).
RNA1     AC-GACGCGUCGAUCCCAAACUCGCAUGAUCCAGACGCC-GCAAUCGGCAC-UG-AGCGAGUGGC--GGCAUCGCCGGCCGG 173
RNA2     AC-GACGCGUCGAUCCCAAACUCGCAUGAUCCAGACGCC-GCAAUCGGCAC-UG-AGCGAGUGGC--GGCAUCGCCGGCCGG 173
RNA3     G--AGACGCGUCGAUUCAAACUUGCAUGACCCAAGCGCCAACUAGCGGCGCUCA-AGCAAGUGGC--GCCAUCGUCGGCCGC 175
SmelC500 AC-GACGCGUCGAUCCCAAACUCGCAUGAUCCAGACGCC-GCAAUCGGCAC-UG-AGCGAGUGGC--GGCAUCGCCGGCCGG 173
RNA4     UCACGACGUGGC------UAGCUCGAUGCA----UCCUCUACCAAGGAGGCGCACGCGAGUUUGGCAGCAUCGCCGGCCGCG 170
ruler    .......110.......120.......130.......140.......150.......160.......170.......180..
```

(b)

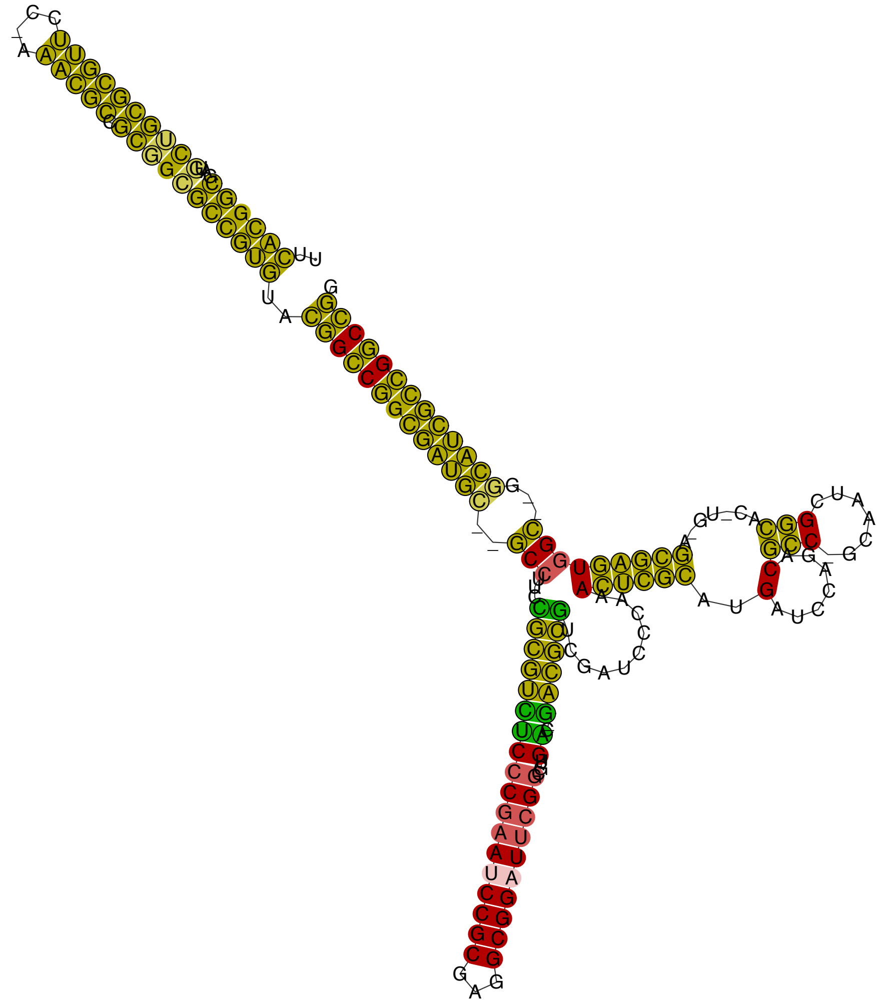

RNA Family Model of SmelC507. (**a**) Alignment of $RFM_{SmelC507}$ sequences (SCI = 0.79); (**b**) Consensus secondary structure (minimum free energy -72.08 kcal/mol) for the alignment shown in (**a**).

(a)

```
          ((.(((((......)))))......(((.(((.((((((((.(((((....))))).))))))))...))).)))..........))..(((((.....(
RNA1      GC-AAUCAGGGAAAUGAUUUCAGUAGCAAUUUAGAUCGGAGCUCGCUUCAGGGUGAACUCCGAUUGCGAGGAUGUACCCGGUCGAGCUCGCGGCGGCCCC  99
RNA2      GC-AAUCAGGGAAAUGAUUUCAGUAGCAAUUUAGAUCGGAGCUCGCUUCAGGGUGAACUCCGAUUGCGAGGAUGUACCCGGUCGAGCUCGCGGCGGC-CC  98
RNA3      GC-AAUCAGGGAACUCAUUUCAGUAGCAAUUUAGAUCAGAGCUCGCUCUACGGUGAACUCUGGUUGCGGGAUUGUACCCGGUUCAGCGCGCGGCGGCUUC  99
SmelC507  GC-AAUCAGGGAAAUGAUUUCAGUAGCAAUUUAGAUCGGAGCUCGCUUCAGGGUGAACUCCGAUUGCGAGGAUGUACCCGGUCGAGCUCGCGGCGGCCCC  99
RNA4      GCAAAUCAUCGAACUGAUUUCAGUAUCAAUUUAGAUCAGAGCUCACUGCGAAGUGAGCUCUGAUU--GAGAAUCGAACCGGUUGAGCUCGCGGCGGCUCU  98
ruler     .........10........20........30........40........50........60........70........80........90.......100

          (.(((((......((((...(.((((((((((.....))).)))))))..))))))))))...).)........((((.......))))))))...
RNA1      CGGGCGGA-GAG-UGAUGGAUA-UGCCCGGUGCGUUUGGCACCCGGGCGUGAUAUUGCCGCC--GG-GAAUAUA-GAGAGAUUGAAGUUUUCGCCGCAAU 192
RNA2      CGGGCGGA-GAG-UGAUGGAUA-UGCCCGGUGCGUUUGGCACCCGGGCGUGAUAUUGCCGCC--GG-GAAUAUA-GAGAGAUUGAAGUUUUCGCCGCAAU 191
RNA3      CGGGCGGA-GAUCUGAUCGAUU-UGCCCGGGCGUUUAGCGA--CGGGCGUGAUAUUGCUGCC--GGUGAAUAU---AGAGAUUGGCAAUUUCGCCGCGAA 190
SmelC507  CGGGCGGA-GAG-UGAUGGAUA-UGCCCGGUGCGUUUGGCACCCGGGCGUGAUAUUGCCGCC--GG-GAAUAUA-GAGAGAUUGAAGUUUUCGCCGCAAU 192
RNA4      CGGGGAAACGUGCUGAUCGGUACAGCCCAGUAUAUUCCAGG-GCGGGCGCGACAUUACCGCCAAAAUGACUGCGCCAUAGGUGGGACAUUUCACCGCUUC 197
ruler     .......110.......120.......130.......140.......150.......160.......170.......180.......190.......200

          ....((((.(((((.((....))))))).)))).....
RNA1      CUACCAGGACCCGCUCGCUGACGGCGGCCCUGUUUUU 230
RNA2      CUACCAGGACCCGCUCGCUGACGGCGGCCCUGUUUUU 229
RNA3      UUUCCAGGACCCGCUCGCUGACGGCGGCCCUGUUUUU 228
SmelC507  CUACCAGGACCCGCUCGCUGACGGCGGCCCUGUUUUU 230
RNA4      CUUGAAGGGCUCGCUUGCUGACAGCGGCCCUUUCUUU 235
ruler     .......210.......220.......230........
```

(b)

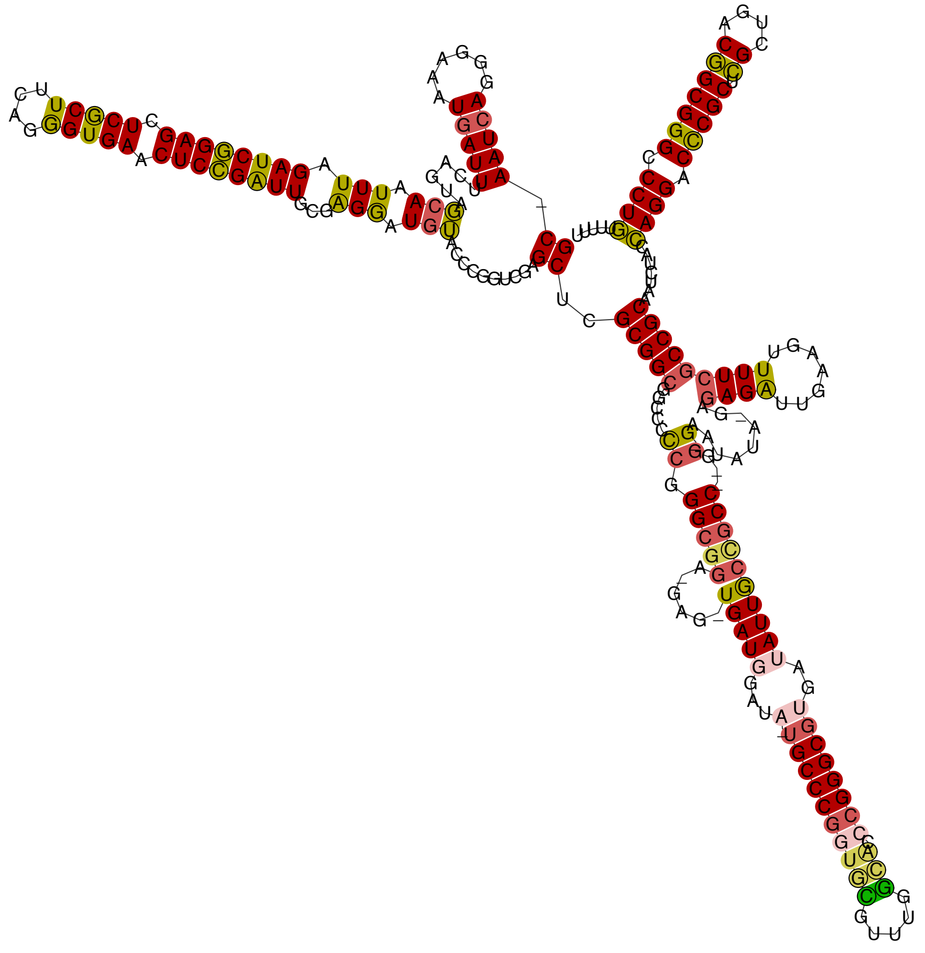

RNA Family Model of SmelC549. (**a**) Alignment of $RFM_{SmelC549}$ sequences (SCI = 0.69); (**b**) Consensus secondary structure (minimum free energy -61.88 kcal/mol) for the alignment shown in (**a**).

(**a**)

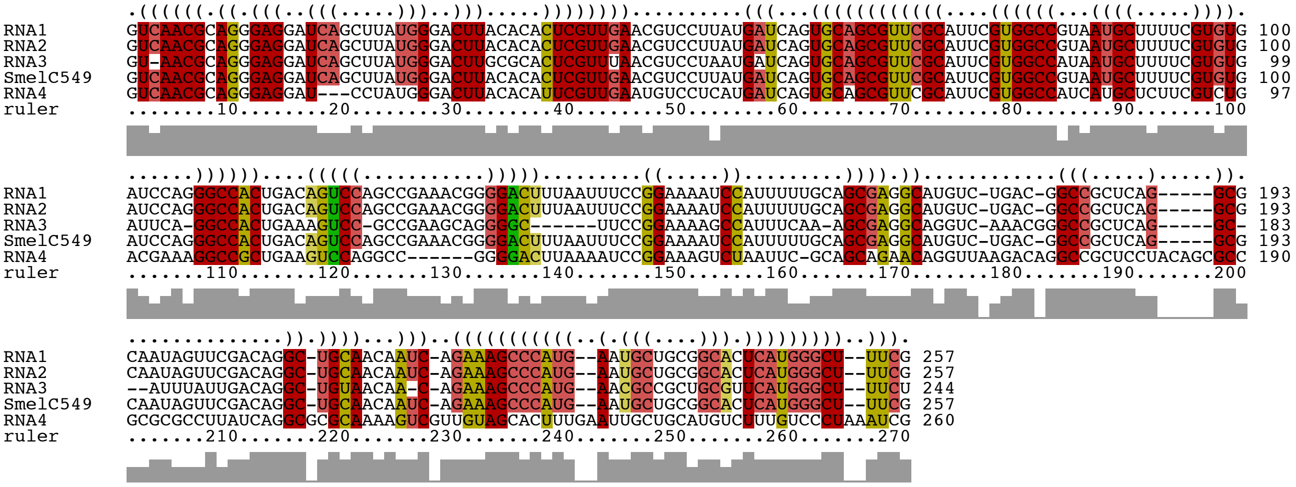

(**b**)

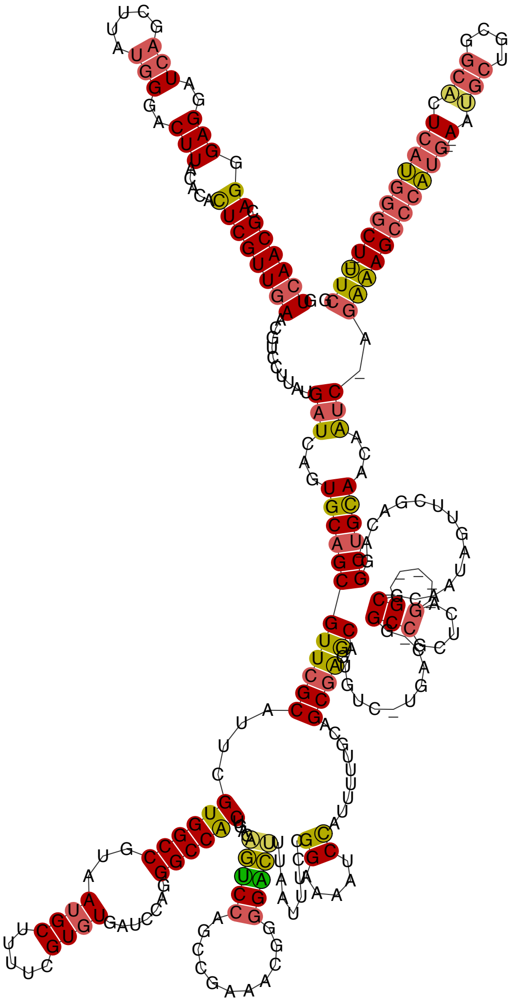

RNA Family Model of SmelC601. (**a**) Alignment of $RFM_{SmelC601}$ sequences (SCI = 0.98); (**b**) Consensus secondary structure (minimum free energy -54.68 kcal/mol) for the alignment shown in (**a**).

(a)

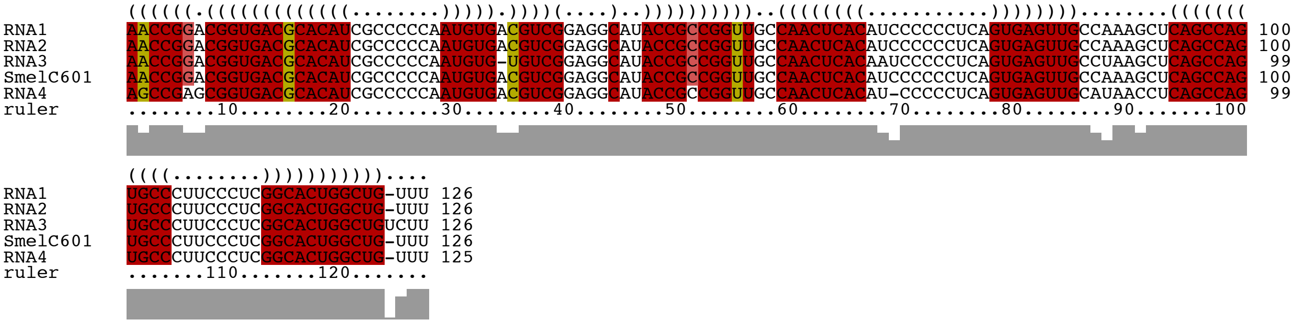

(b)

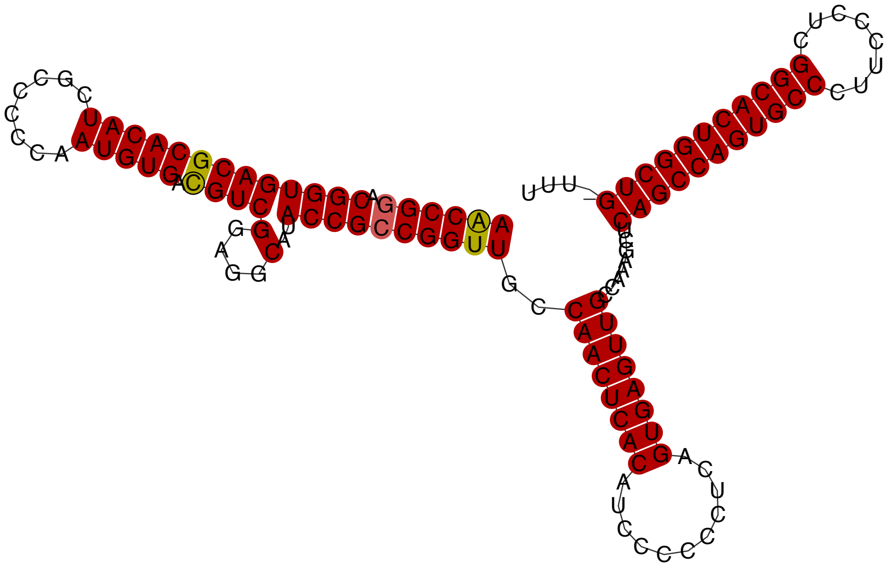

RNA Family Model of SmelC671. (**a**) Alignment of $RFM_{SmelC671}$ sequences (SCI = 0.55); (**b**) Consensus secondary structure (minimum free energy -20.30 kcal/mol) for the alignment shown in (**a**).

(a)

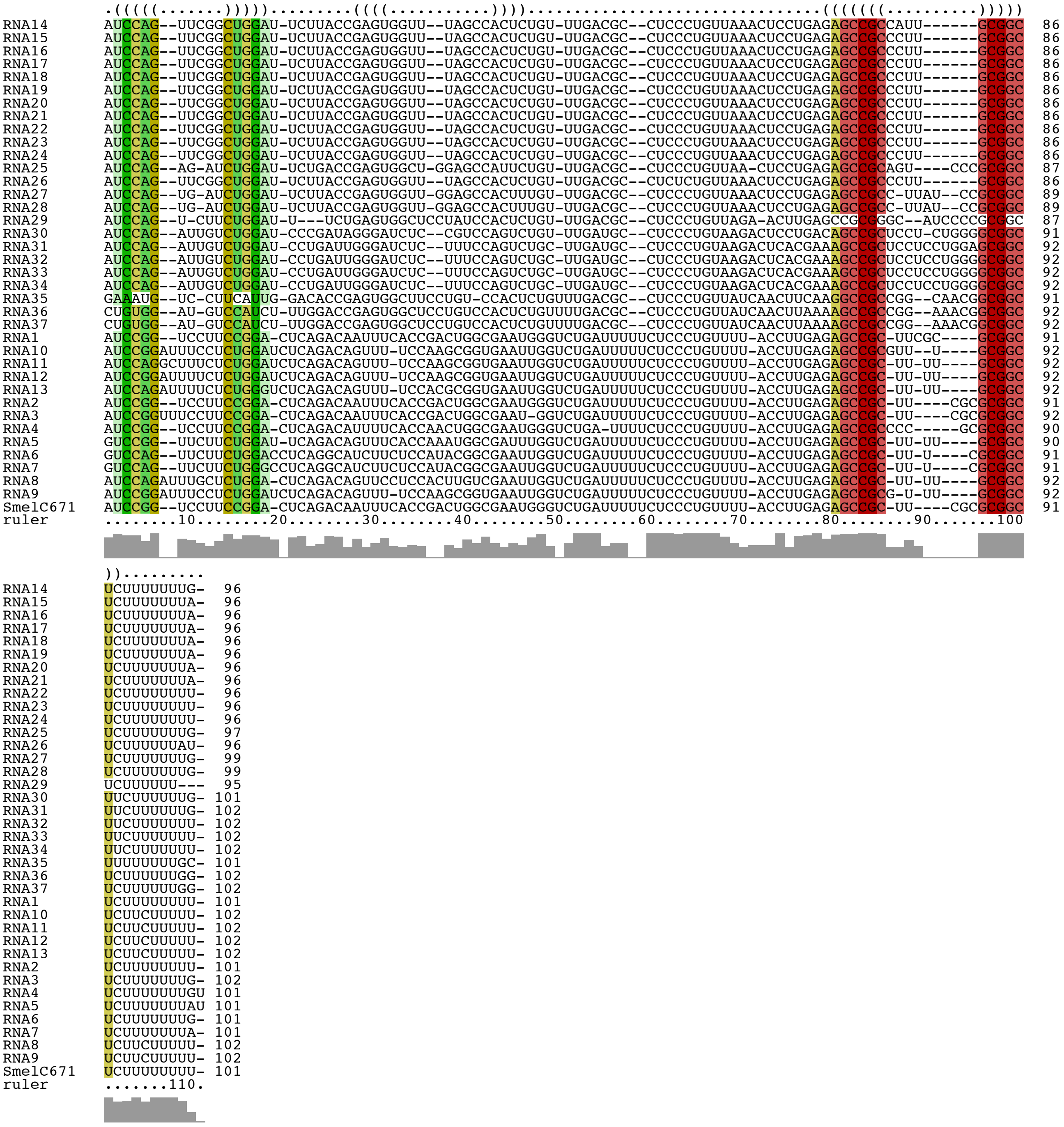

RNA Family Model of SmelC671. *Cont.*

(b)

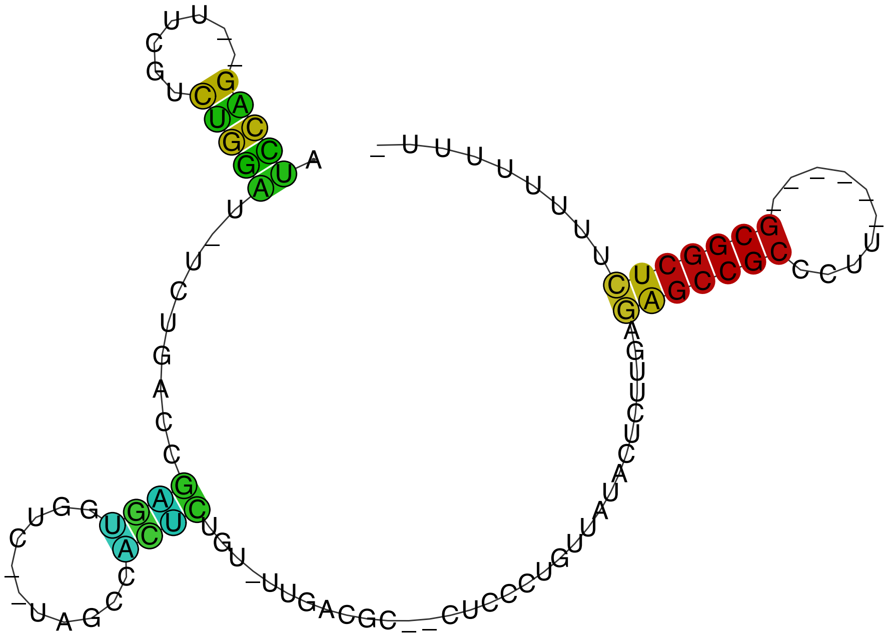

Secondary Structure of Trans-encoded sRNA SmelC749 (minimum free energy -21.30 kcal/mol).

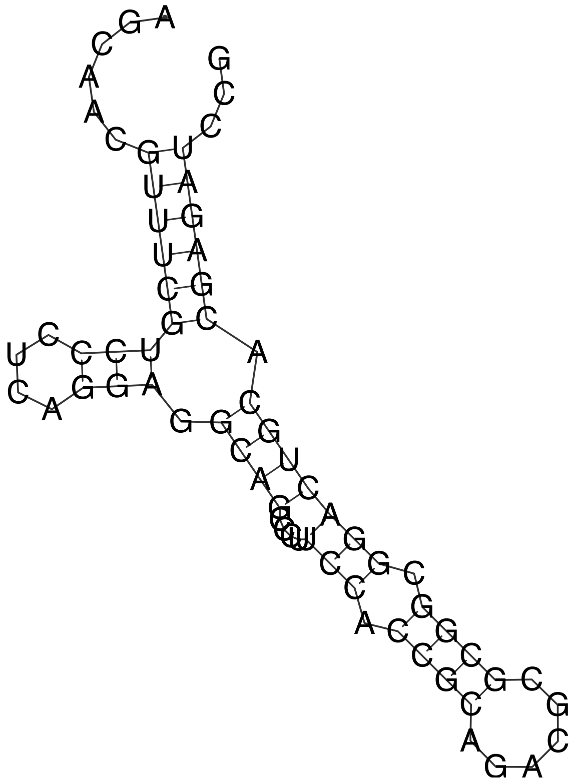

RNA Family Model of SmelC775. (**a**) Alignment of $RFM_{SmelC775}$ sequences (SCI = 1.00); (**b**) Consensus secondary structure (minimum free energy -66.30 kcal/mol) for the alignment shown in (**a**).

(a)

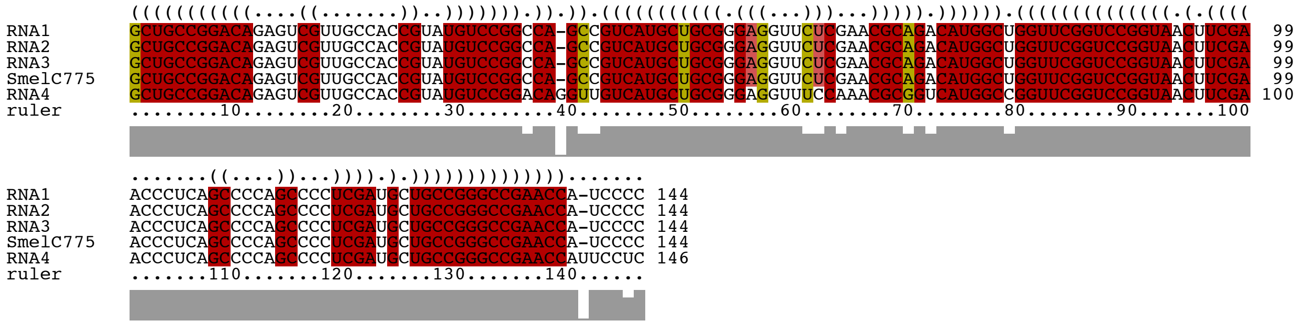

(b)

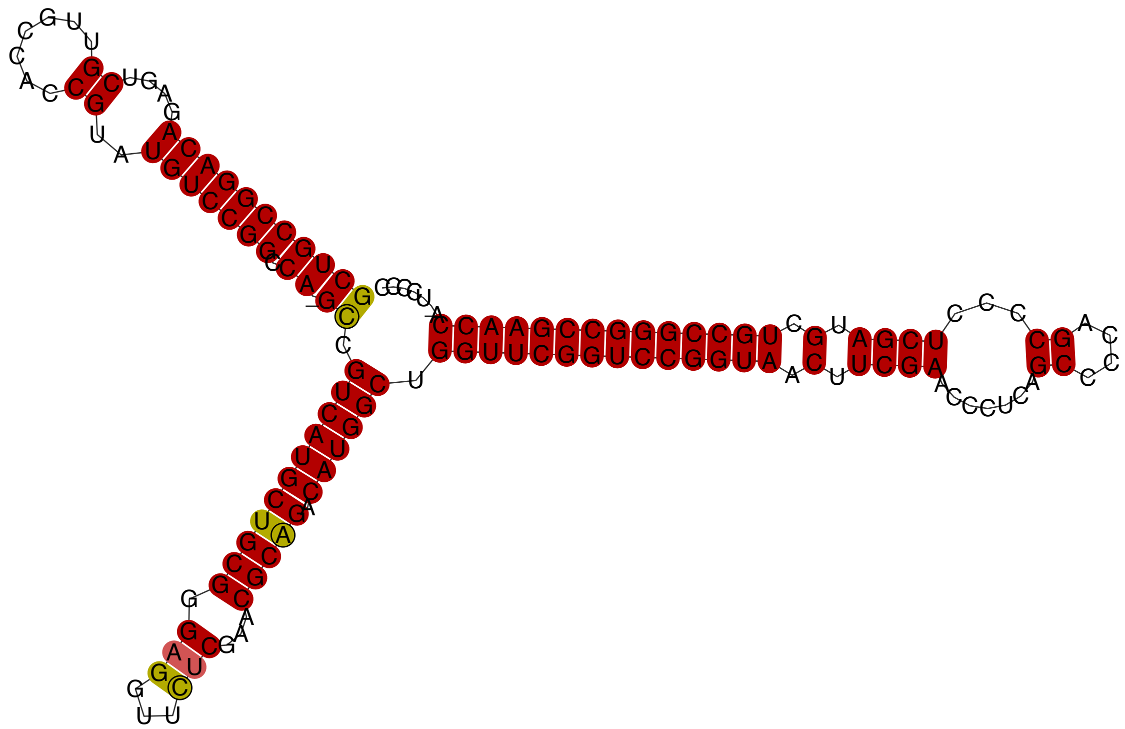

RNA Family Model of SmelC776. (**a**) Alignment of $RFM_{SmelC776}$ sequences (SCI = 0.95); (**b**) Consensus secondary structure (minimum free energy -45.14 kcal/mol) for the alignment shown in (**a**).

(a)

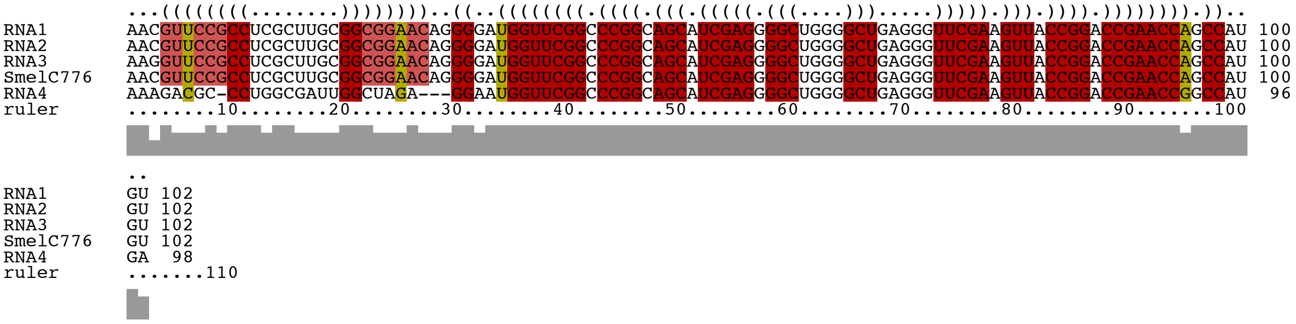

(b)

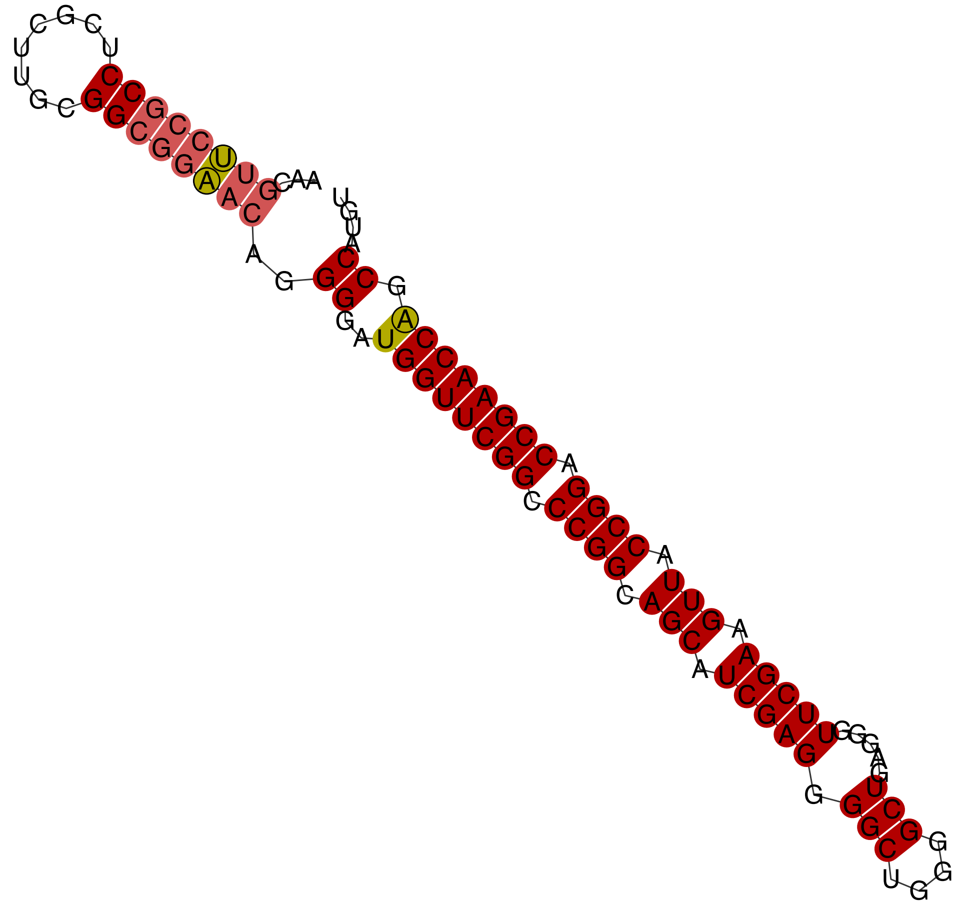

Supplement: Supplementary File 1 — ZIP-Document (ZIP, 882 KB) [file genes-02-00925-s001.zip › genes-11341-supplement-final/genes-11341-Supplement-S1.pdf]
